# The effect of the synchronized multi-dimensional policies on imported COVID-19 curtailment in China

**DOI:** 10.1371/journal.pone.0252224

**Published:** 2021-06-01

**Authors:** Zhenhua Chen, Meng Yu, Yuxuan Wang, Lei Zhou

**Affiliations:** 1 City and Regional Planning, The Ohio State University, Columbus, OH, United States of America; 2 School of Economics and Management, Shanghai Maritime University, Shanghai, China; University of Thessaly, GREECE

## Abstract

As countries are lifting restrictions and resuming international travels, the rising risk of COVID-19 importation remains concerning, given that the SARS-CoV-2 virus could be transmitted unintentionally through the global transportation network. To explore and assess the effective strategies for curtailing the epidemic risk from international importation nationwide, we evaluated “the joint prevention and control” mechanism, which made up of 19 containment policies, on how it impacted the change of medical observation and detection time from border arrival to laboratory confirmation of COVID-19 in its burst in China. Based on 1,314 epidemiological-survey cases from February 29 to May 25, 2020, we found that the synchronized approach of implementing multi-dimensional interventional policies, such as a centralized quarantine and nucleic acid testing (NAT), flight service adjustment and border closure, effectively facilitate early identification of infected case. Specifically, the implementation of the international flight service reduction was found to be associated with a reduction of the mean intervals of diagnosis from arrival to lab-confirmation by 0.44 days maximally, and the border closure was associated with a reduction of the diagnosis interval of imported cases by 0.69 days, from arrival to laboratory confirmation. The study suggests that a timely and synchronized implementation of multi-dimensional policies is compelling in preventing domestic spreading from importation.

## 1. Introduction

**Fig 1:**
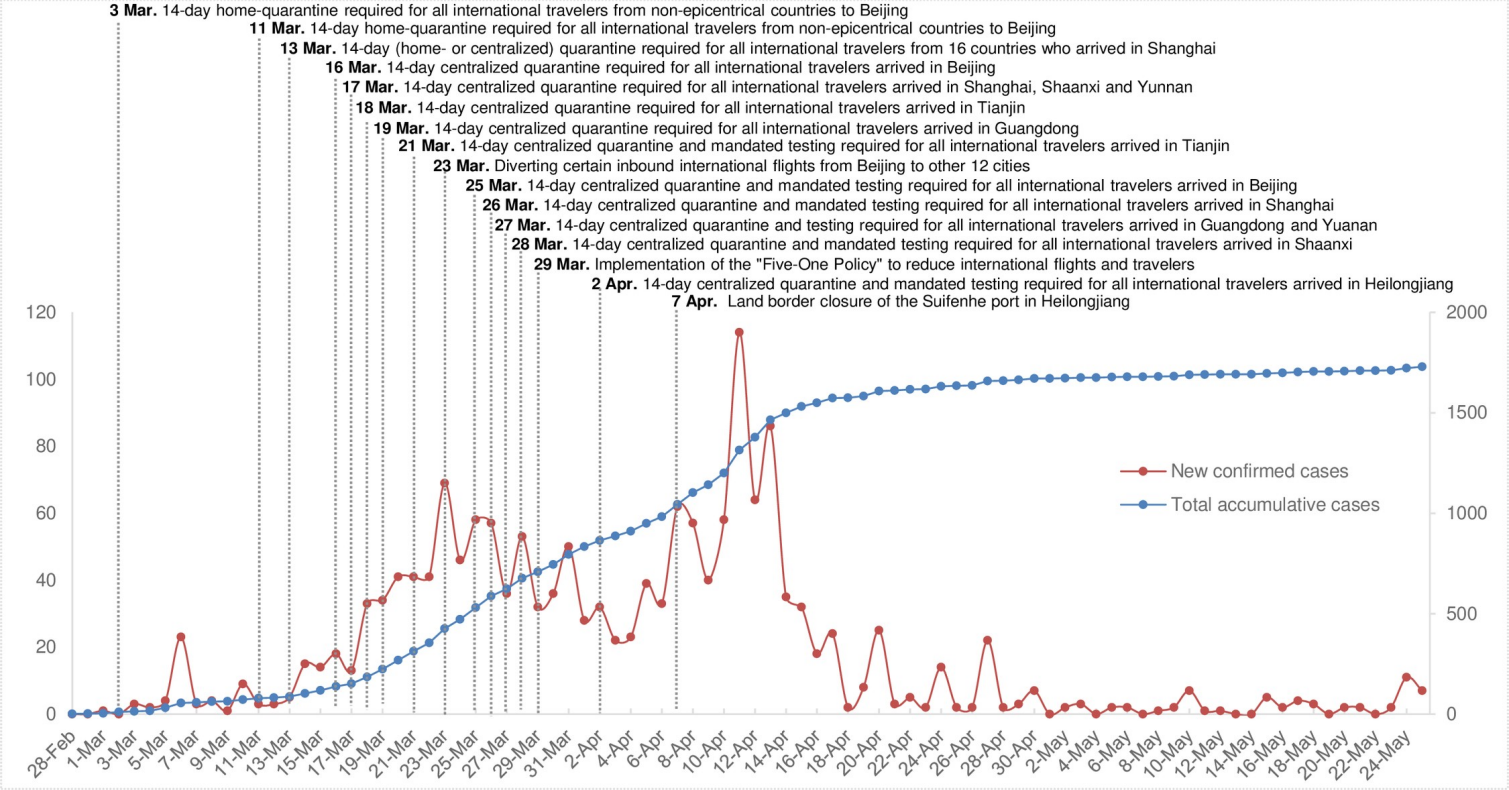
Multi-dimensional interventional policies and the distributions of imported confirmed cases and their diagnosis intervals. The figure illustrates the evolution of abrupt upsurge of COVID-19 imported cases and the progressive multi-dimensional policies implemented in China from February 29 to May 25, 2020 (and no imported case was reported during May 26–31, 2020).

The global public health crisis of the COVID-19 pandemic is far from the end. Since the SARS-CoV-2 virus can be transmitted through the global transportation network [1], and also because the pandemic is more a matter of network interconnectivity than of spatial proximity [2], the challenges of preventing and control for its importation remain pressing. How the cases can be detected effectively so that the connection between importations and domestic communities can be cut off in a timely manner become critical to prevent any local transmission and secondary outbreak [3]. In this paper, we evaluated the effect of a nationwide “joint prevention and control (JPC)” mechanism established by the State Council of China and the multi-faceted policies issued by the JPC on the containment of imported cases. JPC is a multi-ministerial and multi-sectoral coordination working mechanism initiated by the Chinese central government in January of 2020 in combating the domestic outbreak of COVID-19 [4]. The mechanism provides a clear division and responsibility for multi-agencies, including public health, aviation, customs, and logistics, to achieve a collaborative effort for epidemic prevention and control. The JPC mechanism was found successful in terms of curbing the spread of the disease nationwide, preventing the coronavirus from re-entering the country to cause a new epidemic, as well as emphasizing whole-of-government and whole-of-society actions [5, 6]. The assessment focused on the period from the end of February to the end of May in 2020. During this period, the first wave of domestic outbreak, centered in Wuhan, was effectively under control in China, but meanwhile, the country still faced an abrupt upsurge of re-entering COVID-19 cases from overseas hereinafter referred to as imported COVID-19 cases (Fig 1). The research presented the evolution of the 19 key interventions and measures and examined the extent to which these interventional strategies are effective in breaking the chains of transmission and achieving the goal of “Early Detection, Reporting, Isolation and Treatment” [7, 8]. Specifically, this paper measured the variation of the diagnosis time for the imported COVID-19 cases reported by cities in China from the date of port entry to clinical confirmation. Understanding the variation of the indicator is critical as it provides implications for policy interventions to take immediate response to break the channels of transmission through identifying a confirmed case as early as possible. In addition, the marginal effects of each intervention on the variation of the interval were estimated according to when and where it was adopted to evaluate policy’s effectiveness separately and coordinately based on the epidemiological survey on the imported cases in major Chinese cities for the study period.

Our key research finding is that the non-pharmaceutical interventions (NPI), such as quarantine and nuclear acid testing (NAT), flight traffic control and border closure, could only make an impact on speeding up the cases identification process by shortening the diagnosis interval when coordinated and synchronized epidemic emergency response system was established. In addition, the paper also contributes to the practice of COVID-19 prevention and control by providing a holistic empirical evaluation to help the global public health community better understand the evolution of the multi-sectoral response for imported COVID-19 cases control through timely multi-dimensional policies and a wide scale of coordinated implementation. These experiences highlight the importance and necessity of enhancing policy coordination, sharing and mutual learning among countries to achieve an effective JPC of the COVID-19 pandemic.

The paper is organized as follows. Section 2 visits the existing literature to discuss the application scenario and extend the interventional technique to a wider scientific area worldwide. Section 3 discusses our data and measures. Then we demonstrate an empirical time-series model of policies’ effects measurement. Section 4 presents the results and analysis and interpretations of the results, while Section 5 summarizes and concludes.

## 2. Literature review

The existing literature that focuses on policy study on curbing the spreading and transmission of COVID-19, especially in its early outbreak, has extensively examined the effects of different non-pharmaceutical intervention (NPI) measures and policy set on the control of the sources of infection and cutting off the channels of transmission of the SARS-CoV-2 virus, which mainly includes mobility restriction or travel ban [3, 9–12], airport screening [13–15], mandated face-covering [16, 17], social distancing (or rationing social contact) [18–20], lockdowns (or facility closures) [21, 12] and quarantine (or social isolation) [22, 23]. While for better pandemic control that developed by scientific research and clinical expertise [24, 25], many countries have adopted various practices, policies, protocols and guidance of the World Health Organization (WHO) [26].

Evidence from the USA and UK showed that the adoption of a “herd immunity” strategy was not able to prevent the spread of the COVID-19 pandemic [27, 28]. Instead, the severity of coronavirus in terms of mortality rates was found to have surged more rapidly in these countries since the outbreak of the pandemic [29, 30]. In addition, some studies, such as Bonaccorsi et al. (2020) found that the implementation of lockdown in Italy, had an uneven effect on different social groups [12]. Those were affected the most include the poorer population and the municipalities with weaker fiscal capacity. A similar outcome was also found in India, given that a COVID-19 lockdown has left a large number of migrant workers suddenly lost their food and shelter [31]. In the case of Africa, Gilbert et al. (2020) argued that the early evidence indicated that air travel restrictions between key African hub countries and China played a minimal role in suppressing transmission [3]. Instead, as what has been explored from previous contagious diseases, such as MERS and H1N1 pandemic [32, 33], the restrictions only delayed the growing scale and speed of the outbreak. Conversely, some studies, such as Tsiotas and Magafas (2020), showed that the implementations of anti-COVID-19 policies, such as closures of school, business and transportation facilities, could be effective in reducing the COVID-19 infection rate, in the case of Greece [34, 35].

In that context of curbing the transmission by the inbound cases before the adoption of the COVID-19 vaccine, although travel restrictions were found to slow the rate of exportation in some countries, they were still insufficient to contain the global spread of COVID-19[36], since most cases were found to have traveled during the asymptomatic incubation period [10]. In this regard, quarantine is generally considered a key strategy for case screening and isolation in many countries. However, questions, such as who should be quarantined, when the quarantine should be started and which type of quarantine (e.g., self-quarantine, usually at home or centralized quarantine) remains unclear at the initial stage of the imported case surge. In fact, recent studies on COVID-19 exit strategies in Europe suggested that the objective of stringent policies cannot be achieved unless appropriate coordination was implemented [37].

In sum, while the above scholarly efforts have improved our understanding of individual and combined effects of NPI on the prevention and control of virus transmission in a domestic and inter-regional environment, it remains unclear to what extent and under what circumstance the progressive implementations of NPIs by multi-agents was effective in containing the imported COVID-19 cases, particularly in a nationwide context. This paper evaluated a series of interventional policies enabled through the JPC mechanism in China to address this challenge of imported COVID-19 risk control. These policies include early home quarantine, centralized quarantine, mandatory testing, aviation service adjustment and border closure, which were implemented in a progressive and coordinated manner among different cities and regions.

## 3. Materials and methods

As indicated by immunologists, such as Fauci et al. [38], since the pathogenesis of certain carriers, i.e., asymptomatic, can be more complicated than a conventional case, an early diagnosis is essential for the prevention and control of the imported SARS-Cov-2 virus. Hence, to measure the speed of case diagnosis on the imported cases, the period from the date of arrival to the laboratory confirmation (instead of test positivity) of an imported case was adopted in our evaluation. Such a selection has the following two major considerations: First, the outcome tends to be more accurate than that based on the result of NAT given that it reflects a clinical judgment based upon a series of evidence [39], such as clinical symptoms, NAT, chest X-rays, CT scan, and antibody tests [40–42]. In general, we found that the confirmation period lasted relatively longer than that from port arrival to testing positive. Second, the indicator standardizes the measurement of outcomes, which enables us to conduct a reasonable comparison among various cases, including symptomatic onset cases upon arrival, cases developed symptoms during the centralized quarantine period, and asymptomatic cases without symptoms till the end of the quarantine [43].

To assess the impact of each policy and the synchronized effect of the set of policies on the variation of the time of diagnosis for the imported COVID-19 cases in China, we compared the effects of the policies from a temporal perspective according to the time and place of the implementation. The effect of the policy was measured by examining whether its implementation is statistically associated with a reduction in diagnosis interval of the imported cases. A shorter diagnosis interval suggests a rapid identification of the infected case, which hence, may reduce the risk of further transmission. The policies responding to COVID-19 importation in China were implemented sequentially and dynamically in the way that the old policies were replaced with the updated ones with more stringent requirements and countermeasures (e.g. from quarantine to centralized quarantine and to the finally centralized quarantine plus testing). Hence, the interactive effect among the key containment policies was captured and reflected as policies implemented at a later stage that have contained the ones implemented prior to that.

**Fig 2:**
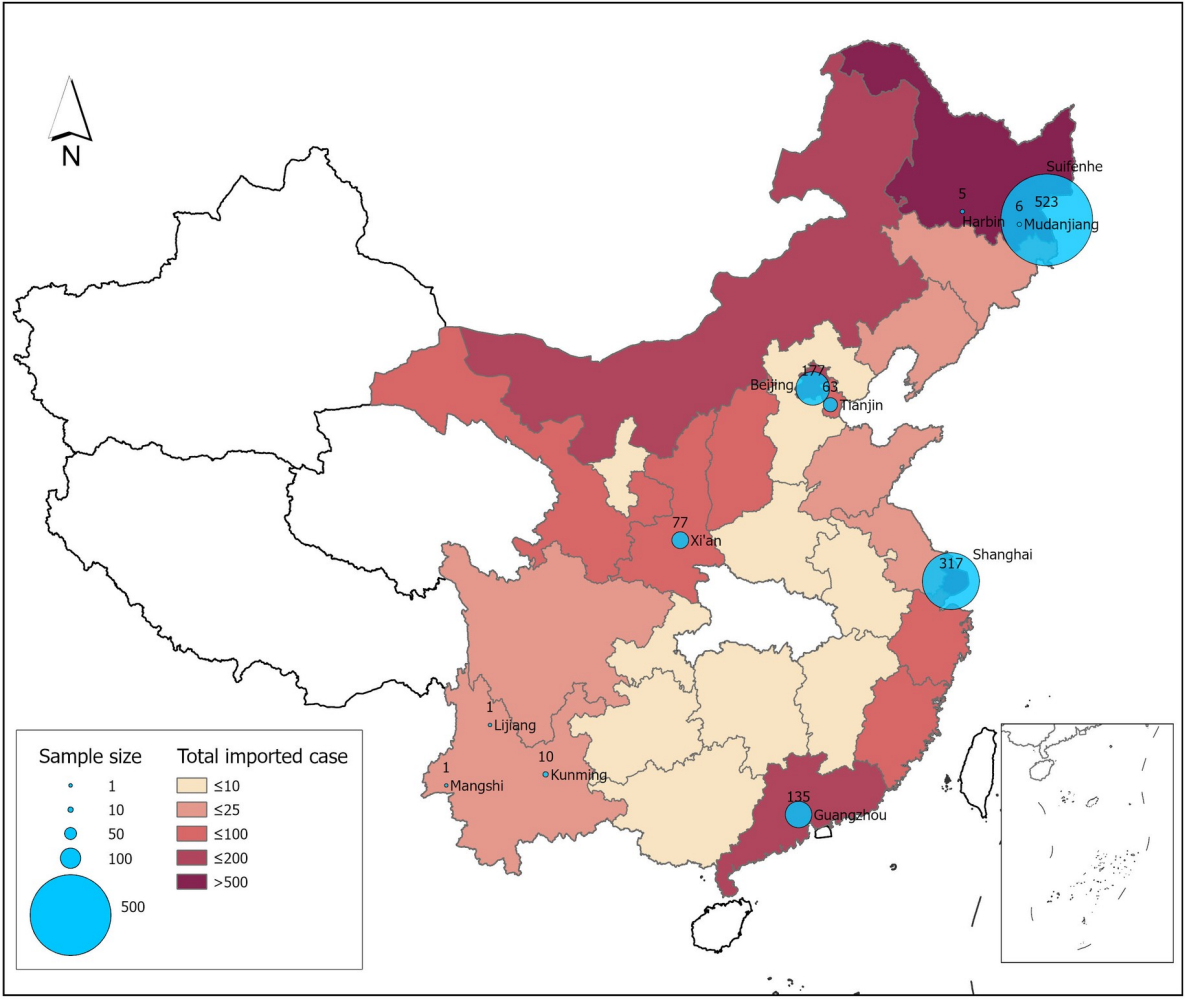
Spatial distribution of imported COVID-19 cases during Feb 29 –May 31, 2020. Note: Among the 1,314 cases we collected, 533 observations are from Heilongjiang province, 174 cases from Beijing, 66 from Tianjin, 317 from Shanghai, 77 from Xi’an, 12 from Yunnan province and 135 from Guangzhou of Guangdong province.

Specifically, the samples from the seven cities and regions with different types of entry ports and at different administrative levels were selected, based on data availability. The sample size is 1,314, which represents the number of imported COVID-19 cases identified through the individual epidemic survey administered by the local centers of disease control (CDC). As shown in Fig 2, Beijing, Tianjin and Shanghai are the three large municipalities, which located along the north and east of China. In particular, the latter two cities have both air and seaport of entries. Although the capital city Beijing only has an airport of entry, it essentially connects with 59 countries and regions [44]. The imported cases identified at Guangzhou and Xi’an were included to capture the inbound passengers from the south gate of China and receive the diverted international flights from Beijing, respectively. In addition, the imported cases from Heilongjiang province (in the northeast) and Yunnan province (in the southwest) were also included. The former is connected with Russia via land ports (e.g., Suifenhe), and the latter has a land connection with multiple South Asian countries (Myanmar, Laos and Vietnam). One should note that given that China does not have a large volume of international passengers who arrived through the seaport, even under a normal circumstance, as the majority of international passengers arrived in China via either airport and land port, no confirmed cases were found to have entered via seaport in our sample.

In terms of the policies for the evaluation, 19 key interventional policies, which were initiated by different ministries and implemented by different provincial and municipal governments under the JPC mechanism, were classified into three categories, including quarantine and NAT, international flight service adjustment (including service cut and diversion), and border closure. The first category of policies was implemented by local health committees, whereas the latter two categories were administered by the General Administration of Civil Aviation of China (CAAC) and the General Administration of Customs (S1 Table in S1 Appendix).

We also examined the statistical association between implementations of policies and the variation of diagnosis interval, both individually (following a chronological order) and collectively, to measure the individual effect and the synchronized effects of policies, using a fixed effect negative binomial model (FENB).

### 3.1. The panel dataset

Data used for the analysis were drawn from different sources. During February 28—May 31 2020, the accumulated imported COVID-19 confirmed cases nationwide were 1,756 and distributed among 26 provinces and municipalities, suggesting that our sample accounts for 74.8% of the population in the same period. The following sections introduce the specifics of the data used for the statistical regression analysis. Details of the data are provided in S2 Table in S1 Appendix.

### 3.2. COVID-19 epidemiological survey data

Started on January 26 2020, the JPC mechanism of the State Council required that all newly arrived international travelers must complete a health declaration form and epidemiological survey at the custom border inspection at the port of entries in China. Information collected from the epidemiological survey includes detailed sociodemographic information of the traveler, clinical symptoms and travel histories.

The data of all the confirmed cases were reported and updated daily on the websites of local municipal health commissions. All the published records of the confirmed imported COVID-19 cases reported by the seven cities and provinces for the period Feb 29 –May 31, 2020 were collected. Among the 1,314 cases, 533 observations (40.47% of the sample) are from Heilongjiang province, 174 cases from Beijing (13.24% of the sample), 66 (5.02% of the sample) from Tianjin, 317 (24.12% of the sample) from Shanghai, 77 (5.86% of the sample) from Xi’an, 12 (0.91% of the sample) from Yunnan province and 135 (10.27% of the sample) from Guangzhou of Guangdong province. Information from the epidemiological data was adopted to develop the dependent variable *diagnosis interval* and independent variables, which includes *asymptomatic case*, *symptomatic onset upon arrival*, *country of original departure*, *transfer or not* and *travel purpose*.

In addition, given that at the early stage of the pandemic, not many countries had implemented strict containment policies, we thus assumed that there was a correlation between an imported confirmed case and the original country of departure. To control for the level of strictness of the COVID-19 containment policies in different nations, we adopted the daily index of the COVID-19 government response tracker developed by the University of Oxford (https://covidtracker.bsg.ox.ac.uk) as *policy stringent level*.

### 3.3. Travel data

In addition, we collected the real-time data of international flight services connecting all the origins and transfer cities with the Chinese cities for the same period from Flightradar24.com, a global flight tracking service company. We first validated the flight information, such as origin, destination, date of departure & arrival, flight duration (in hours), aircraft model, seat capacity, and service status, based on the official flight inquiry website of CAAC, and then matched with all the confirmed cases. Similarly, the schedule information of intercity bus connecting China’s border city Suifenhe and cities in Russia (such as Vladivostok and Ussuriysk) where travelers took cross-border bus were collected from www.rome2rio.com. The information above was used to establish key independent variables, such as *trip length*, and *the number of travelers associated with each confirmed case* to capture travel characteristics.

### 3.4. NPI-policy variables

As showed in Fig 1 (a detailed explanation was summarized in S1 Table in S1 Appendix), the different NPI policies were measured in dummy variables according to the time and place of implementation. Since some policies were only implemented for a certain time period while others were implemented till the end of the study period, we created policy variables based on the “time range” (represented by “*r*”) of the interventions to avoid an overlap of their interactions. For instance, variables, such as *p311bjr*, *p313shr*, *p316bjr*, *p317shr*, *p317ynr*, *p317xar*, *p319gdr* represent that these policies were only implemented for a limited time period, while other policies, such as *p321tj*, *p325bj*, *p326sh*, *p327yn*, *p327gd*, *p328xa*, *p402hlj* and *p407hlj*, were implemented continuously since its introduction. In addition, we also removed variable that either has a strong serial correlation or has no variation. The variables reflecting flight adjustment (*pfl*) and re-routing (*pfr*) were coded in different dummy variables to represent the different phases or cities with an adjusted service. For instance, *pfl-1* represents the initial stage of flight adjustment, which is also the baseline. *pfr-1* represents the city of Beijing, in which part of international flights was rerouted to other cities. *pfr-2* stands for the cities of Tianjin, Shanghai and Xi’an, all of which received rerouted flights from Beijing.

### 3.5. Other control variables

In addition, a wide range of location characteristics that might influence importation cases were introduced as the control variables. Specifically, such characteristics include socioeconomic status, which is represented by variable *GDP per capita*, the administrative hierarchy, which is classified as the *administrative level*, geographical locations, which is represented by *Border type*, as well as *local daily new confirmed cases* which is a proxy of other local policies for virus control enacted in parallel. One should note that we did not control domestic flights because the guideline for international inbound airlines released by the International Civil Aviation Organization (ICAO) has been strictly executed by all the airports in China to prevent the spread of COVID-19 [45]. Another key reason is that domestic flights and international flights were regulated and operated in a very different procedure. Hence, we assumed the influence of domestic flight operation had a negligible influence on the operation of international flights.

### 3.6. Methodology

Our rationale to measure the effect of NPI-policies on COVID-19 risk control is to examine whether diagnosis intervals decline as multiple intervention policies were implemented, cumulatively and progressively, in different cities and provinces. The dependent variable reflects the number of days (or the time interval) of each case from the date of his/her arrival to the date of COVID-19 lab confirmation. The distribution of the dependent variable *Y_it_* is illustrated in S1 Fig in S1 Appendix. Given that the issue of overdispersion (as shown in S3 Table in S1 Appendix), a fixed-effect negative binomial (FENB) model was adopted for the statistical analysis to control for the unobserved variables that are constant over time, and meanwhile generalize the Poisson regression model by introducing a dispersion parameter to relax the equi-dispersion restriction of the Poisson model.

While it is generally recognized that FENB allows for individual-specific variation in the dispersion parameter rather than in the conditional mean, it does not control for time-invariant covariates because these invariant covariates usually generate non-zero coefficient estimates, which may lead to spurious statistical significance [46–48]. As a feasible solution, Guimarães proves that if the estimation includes at least 20 variables per individual in the panel as large as 1000 individuals, the type I error, which is a "false positive" finding, can be adequately controlled for [48]. Since our panel data has 1,314 individuals with 79 variables per individual (14 key explanatory variable, 17 policy variables, and 48 dummy variables representing different countries of origins), we are good to use the FENB. The following equation describes the whole model based on the log-likelihood function of the negative binomial distribution.

$$log(Y_{it})=\beta_{0}+\beta_{1}x_{it}+\gamma Z_{i}+{Time}_{t}+\epsilon_{it}$$ (1)

$$x_{it}\in \{COVID-19\;symptoms,Travel,NPI-Policies\}$$

$$Z_{i}\in \{GDP_{PC},Administrative\;level,Border\;type,Local\;new\;confirmed\;cases\}$$

The dependent variable *Y_it_* represents the expected log count of the diagnosis interval for each case observed in city (or province) *i* during *t*. *x_it_* represents a series of variables, such as COVID-19 symptom-related characteristics, travel status that affiliated with each case observed in city (or province) *i* at *t* period, a stringent policy which measures government responses to COVID-19 at different countries of origins, and the interventional policies implemented associated with each case *Z_i_* represent the city- (or provincial-) level time-variant control variables, including city (or provincial) GDP per capita, administrative level, border type, and *Time*_t_. *t* stands for the time period-specific effect in the period. To provide a clear interpretation of the estimated outcomes, the average marginal effects at means (MEM) of diagnosis interval were reported using econometric packages of Stata.

Specifically, we assumed that the variation of the time interval of cases from the date of port entry to the date of clinical confirmation could be explained by four groups of variables, including (i) COVID-19 symptom-related characteristics, (ii) travel-related information, (iii) the NPI-policy related influences, and (iv) the local controls. The symptom-related characteristics include dummy variables, representing whether a case is asymptomatic and whether she/he has a symptom onset upon arrival. The travel-related information includes both dummies and continuous variables that represent the length of the trip (in hours), whether a trip involves a transfer or not, whether a trip is either tour-related, study-related, resident-related, or work-related. In addition, we also controlled for the number of travelers who arrived in city (or province) *i* on the same day, which was estimated based on the number of inbound flights and intercity buses in terms of the corresponding flight and bus seat capacity. The departure country of origin in the forms of dummy variables as well as the daily policy stringency’s level in the departure country was also introduced to control the heterogeneous effect of risk situations in different countries.

All independent variables were examined carefully to avoid potential multicollinearity and the modeling structure with the most robust outcomes was identified (S4A–S4C Table in S1 Appendix). In the end, 17 NPI-policy-related dummy variables were introduced based on the specific affected cases, the city and the date of their corresponding implementations.

The analysis was conducted in the following steps. First, we fitted the model by introducing each NPI-policy variable sequentially into the model, following a chronological order of the date of its implementation, while controlling for the group variables (i), (ii) and (iv). The advantage of this approach is that both phasic effects and synchronized effects of the 17 NPI policies can be examined as they were introduced respectively in parallel with the explanatory variables of other groups (S5 and S6 Tables in S1 Appendix). According to Table 1, the results show that the variations of group (iii) effects, the NPI-policy variables, show phase characteristics over time, ceteris paribus, which demonstrates the various effects of models in validating the policies effects. Lastly, the MEM of all the variables was estimated.

**Table 1:** Marginal effects of the time interval of case confirmation to the synchronized effect of multi-dimensional policies with multiple controls.

**Model ID**	**1**	**2**	**3**	**4**	**5**	**6**	**7**
**Variable name**	**11Mar**	**13Mar**	**16Mar**	**17Mar**	**19Mar**	**pfl**	**21Mar**
3.11, Quarantine policy, Beijing	0.30	0.31	0.45	0.41	0.41	0.43	0.45
3.13, Quarantine policy, Shanghai		0.24	0.26	0.25	0.25	0.17	0.16
3.16, Upgraded quarantine policy, Beijing			0.30	0.26	0.26	0.19	0.21
3.17, Updated quarantine policy, Shanghai				0.08	0.08	-0.06	-0.06
3.17, Updated quarantine policy, Yunnan				1.70***	1.70***	1.64***	1.63***
3.17, Updated quarantine policy, Xi’an				0.74**	0.74**	0.61*	0.61**
3.19, Quarantine policy, Guangdong					0.04	-0.11	-0.11
3.19, Flight reduction: Phase 2						0.04	0.04
3.21, Quarantine & Testing, Tianjin							0.46
3.23, Flight diversion, Beijing							
3.23, Receiving flights rerouted from Beijing							
3.25, Quarantine & Testing, Beijing							
3.26, Quarantine & Testing, Shanghai							
3.27, Quarantine & Testing, Yunnan							
3.27, Quarantine & Testing, Guangdong							
3.28, Quarantine & Testing, Xi’an							
3.29, Flight reduction -phase 3 (the "Five-one")						-0.24	-0.24
4.02, Quarantine & Testing, Heilongjiang							
4.07, Border closure, Heilongjiang							
Transfer or not (1 = transfer, 0 = direct)	0.25***	0.24**	0.24***	0.24**	0.24**	0.19**	0.20**
Asymptomatic (1 = asymptomatic, 0 = symptomatic)	-0.19***	-0.19***	-0.19***	-0.19***	-0.19***	-0.18***	-0.18***
Symptomatic onset case upon arrival (1 = symptomatic, 0 = no symptoms)	-0.57***	-0.56***	-0.56***	-0.58***	-0.58***	-0.57***	-0.58***
No# of travelers associated with each confirmed case	-0.03	-0.03	-0.03	-0.04	-0.04	-0.04	-0.04
Administrative level: 2 (1-provincial capital; 2-municipality/province)	-2.15***	-2.11***	-2.13***	-2.29***	-2.28***	-2.46***	-3.13***
GDP per capita (10,000 USD)	0.57***	0.57***	0.57***	0.58***	0.58***	0.62***	0.85**
Border type: 2 (1 = air, 2 = air+land)	2.06***	2.04***	2.13***	2.03***	2.03***	2.31***	3.06**
Border type: 3 (1 = air, 3 = air+harbor)	0.87***	0.86***	0.95***	0.88***	0.88***	0.96***	0.99***
Policy stringency level	-0.01***	-0.01***	-0.01***	-0.01***	-0.01***	-0.01***	-0.01***
N = observation	1176	1176	1176	1176	1176	1176	1176
**Model ID**	**8**	**9**	**10**	**11**	**12**	**13**	**14**
**Variable name**	**pfr**	**25Mar**	**26Mar**	**27Mar**	**28Mar**	**2Apr**	**7Apr**
3.11, Quarantine policy, Beijing	0.44	0.42	0.40	0.36	0.34	0.46	0.46
3.13, Quarantine policy, Shanghai	0.17	0.17	0.59*	0.59*	0.59*	0.56*	0.56*
3.16, Upgraded quarantine policy, Beijing	0.21	0.15	0.23	0.27	0.25	0.19	0.20
3.17, Updated quarantine policy, Shanghai	-0.06	-0.06	0.36**	0.41**	0.40**	0.36**	0.36**
3.17, Updated quarantine policy, Yunnan	1.63***	1.63***	1.61***	1.59***	1.60***	1.45***	1.44***
3.17, Updated quarantine policy, Xi’an	0.60*	0.60*	0.44	0.35	-0.11	0.20	0.20
3.19, Quarantine policy, Guangdong	-0.11	-0.11	-0.07	0.14	0.13	-0.05	-0.04
3.19, Flight reduction: Phase 2	0.04	0.04	0.04	0.00	0.01	-0.07	-0.06
3.21, Quarantine & Testing, Tianjin	0.47	0.45	0.32	0.40	0.51	0.58	0.59
3.23, Flight diversion, Beijing	-0.10	0.04	0.12	0.16	0.16		
3.23, Receiving flights rerouted from Beijing	-0.01	-0.01	0.18	0.25*	0.29*		
3.25, Quarantine & Testing, Beijing		-0.69	-0.69	-0.76	-0.79	-0.73	-0.73
3.26, Quarantine & Testing, Shanghai			0.79***	0.92***	0.91***	0.56***	0.57***
3.27, Quarantine & Testing, Yunnan				0.07	0.07	-0.36	-0.39
3.27, Quarantine & Testing, Guangdong				0.35	0.34	0.04	0.05
3.28, Quarantine & Testing, Xi’an					-0.48	-0.25	-0.25
3.29, Flight reduction -phase 3 (the "Five-one")	-0.25	-0.24	-0.36**	-0.44***	-0.41**	-0.44***	-0.42***
4.02, Quarantine & Testing, Heilongjiang						-0.64***	-0.59***
4.07, Border closure, Heilongjiang							-0.69***
Transfer or not (1 = transfer, 0 = direct)	0.20**	0.20**	0.21**	0.22**	0.21**	0.24**	0.23**
Asymptomatic (1 = asymptomatic, 0 = symptomatic)	-0.18***	-0.18***	-0.17***	-0.17***	-0.18***	-0.15***	-0.18***
Symptomatic onset case upon arrival (1 = symptomatic, 0 = no symptoms)	-0.57***	-0.58***	-0.59***	-0.58***	-0.59***	-0.62***	-0.62***
No# of travelers associated with each confirmed case	-0.04	-0.03	-0.02	-0.01	-0.01	-0.02	-0.02
Administrative level: 2 (1-provincial capital; 2-municipality/province)	-3.12***	-3.08**	-2.69**	-2.65**	-3.03**	-2.93**	-2.92**
GDP per capita (10,000 USD)	0.85**	0.83**	0.56	0.57	0.66	0.69	0.68
Border type: 2 (1 = air, 2 = air+land)	3.04**	2.97**	2.50*	2.66**	2.85**	3.28**	3.36**
Border type: 3 (1 = air, 3 = air+harbor)	0.97***	0.93***	0.60	0.56	0.49	0.61	0.60
Policy stringency level	-0.01***	-0.01***	-0.01***	-0.01***	-0.01***	-0.01**	-0.01**
N = observation	1176	1176	1176	1176	1176	1176	1176

*P<0.1

**P < 0.05

***P < 0.001; n = 1,177 (effective observations).

a. The margins represent the average marginal effect which shows the level change (in terms of a number of days) before and after the implementation of a certain policy, while keeping other variables at means.

b. The dummy variables that represent the country of origins were shown in Model 5 in S1 Appendix.

## 4. Results and discussion

We estimated the change in marginal effects with a focus on a cumulation of policies, which illustrates the mean diagnosis time variance (measured in the number of days) either reduced or prolonged over times and across cities. The key regression results are shown in Table 1, with comprehensive results presented in S5 Table in S1 Appendix.

### 4.1. The effects of travel tracing via epidemiologic survey

**Fig 3:**
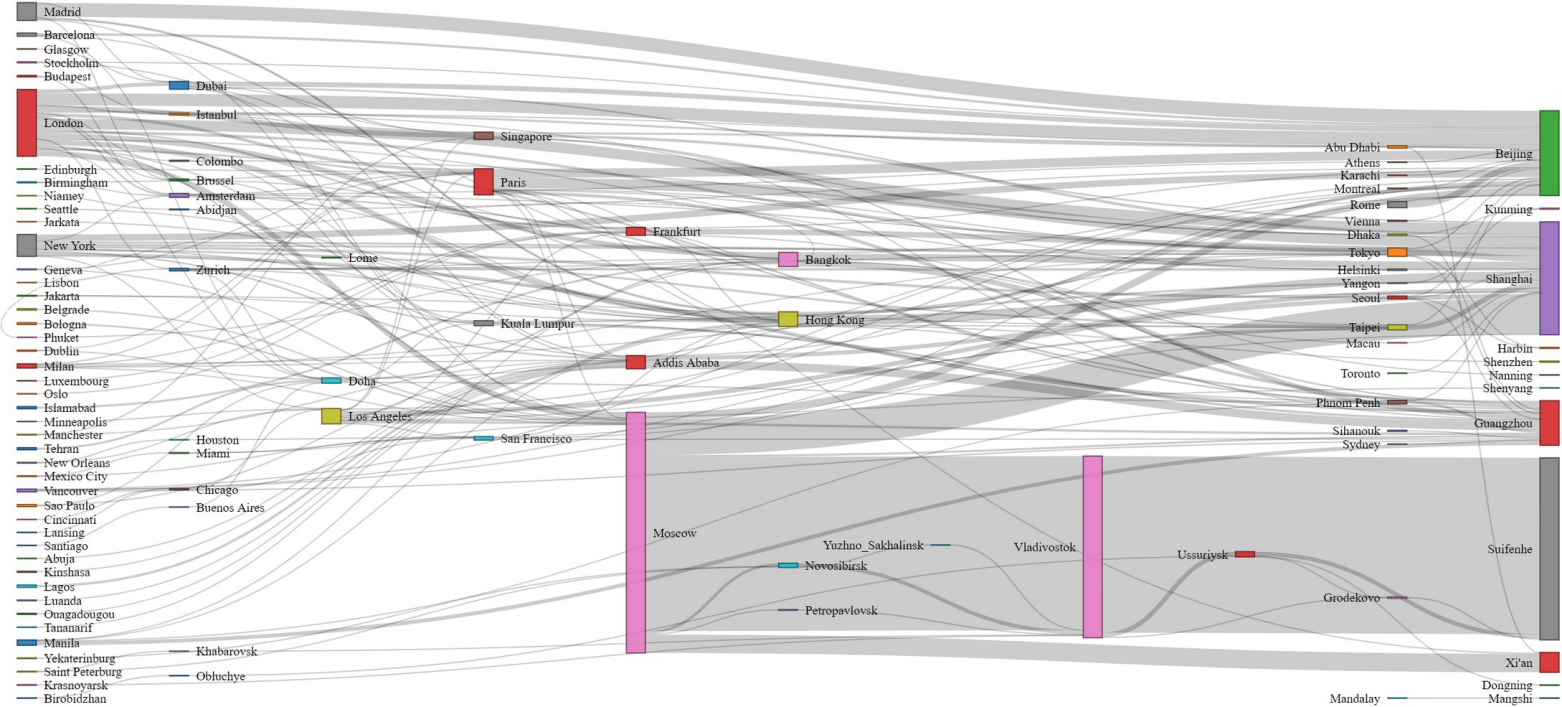
Inbound travel routes of 1,237 imported cases during February 29 –May 31, 2020. Note: Based on the sample data, 189 cases were reported traveling from Europe and the U. S. in the early outbreaks of COVID-19, mainly from London (119), New York (42), and Paris (28). Along with the reduction of the international flights started on March 29 and onward, most imported cases (679) were reported that either originated from or transferred via Russia.

**Fig 4:**
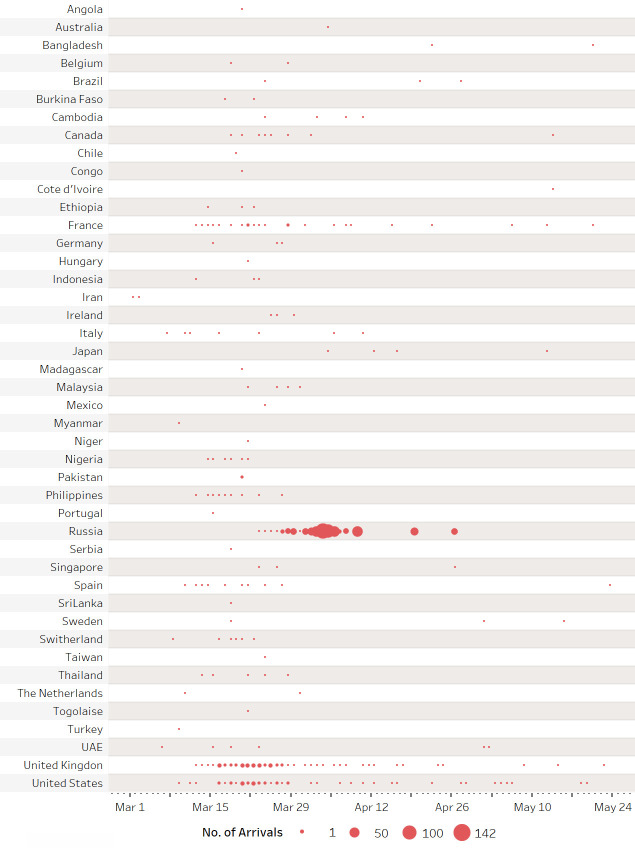
Temporal change of imported case by countries during February 29 –May 31, 2020. Note: The summary was based on the date of arrival. The number of importations from Russia is relatively concentrated and large, while the ones from the severely affected countries such as the U.K., the U.S. and France lasted longer.

Figs 3 and 4 illustrate the spatial and temporal distributions of the confirmed cases based on their specific travel information, respectively. Fig 3 demonstrates important international transfer hubs include Paris, Frankfurt in Europe, Hong Kong and Bangkok in Southeast Asia, Addis Ababa in Africa, as well as Vladivostok in Russia (these cities accounted for 62% of all transfers in our sample). The statistical analysis reveals that the diagnosis interval of the confirmed imported cases with a transfer is about 0.2 days longer than those without a transfer. Fig 4 shows that the density distribution in the timeline of the imported cases based on their origins. It is clear that the peak of the COVID-19 importation was between the end of March and early April. In terms of the distribution by origin, almost 52% (or 685 cases) of the samples were from Russia. Prior to the period, a surge of inflow of cases occurred during mid- and the end of March, was mainly from the United Kingdom (203 cases, or 15% of the sample) and the United States (116 cases, or 9% of the sample). The regression results (as shown in Table 1) show that travelers from Russia are associated with a prolonged diagnosis interval by 0.32–0.48 days, while travelers from the United Kingdom are found to be associated with about 0.2 days less than others in the interval. Such a result revealed a strong heterogeneous characteristic of the diagnosis interval among various cases.

**Fig 5:**
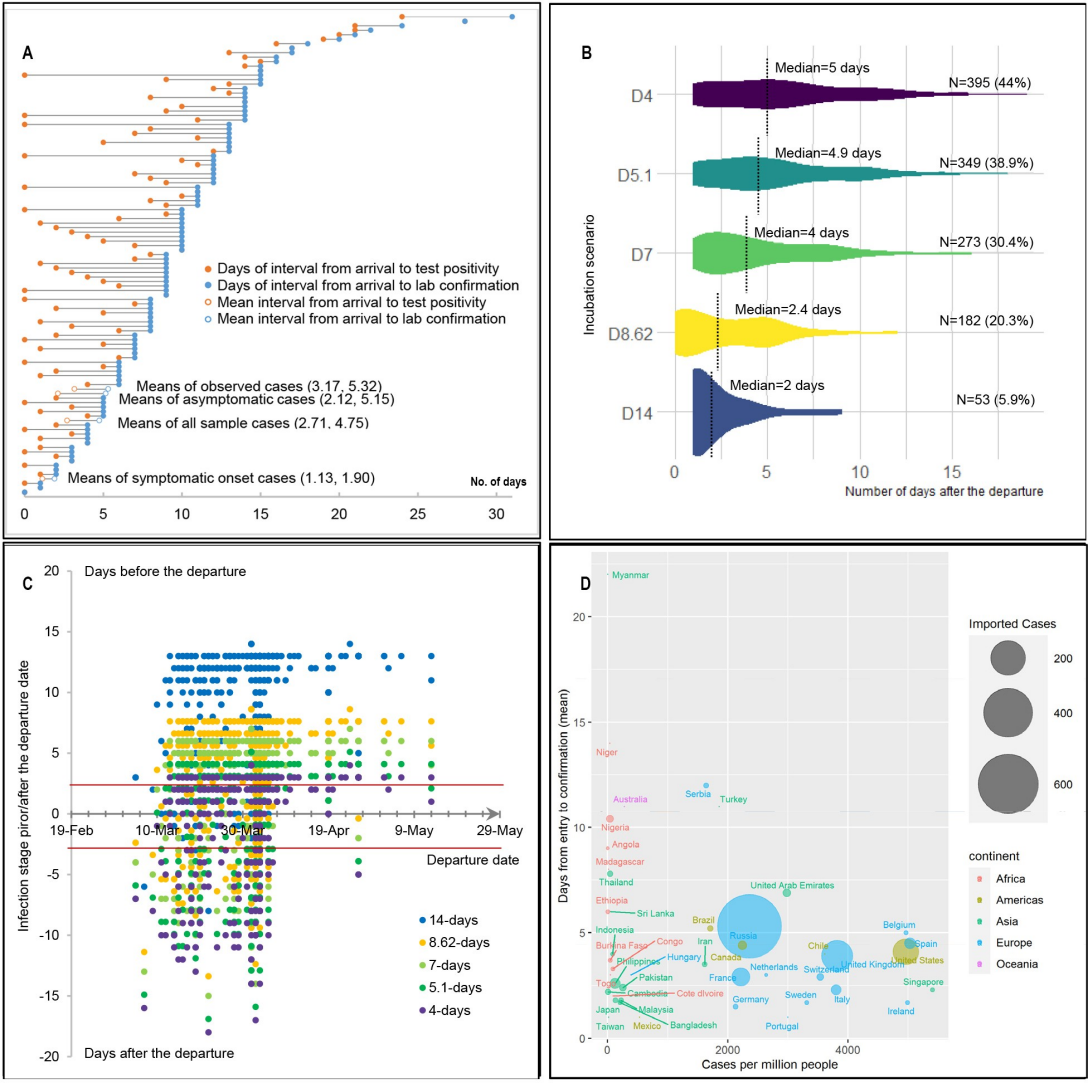
Characteristics of the imported COVID-19 cases. (A) reveals the distributions of the periods from the port of entry to testing positivity and lab confirmation, respectively, based on the sample data. The mean interval from arrival to test positivity and to a lab confirmation of the four groups of cases are shown in the parenthesis. (B) illustrates the time interval from entry to a case confirmation and the sample distribution based on the assumption of different incubation periods. (C) reflects the estimated temporal distribution of possible infection dates of the samples, based on the date of departure, date of confirmation, and assumptions of different incubation periods. (D) shows the correlation between the average time of confirmation of cases and the epidemic risk of different countries.

In addition, our results show (Table 1) that the diagnosis interval of the asymptomatic case (221 identified through NAT, or 16.8% of the sample) and the cases with a symptom onset upon arrival (186 identified through self-reporting, or 15.6% of the sample) are consistently lower by 0.2 days and 0.62 days than other cases, respectively. Such a result confirms that epidemiological survey information is essential for customs and medical workers to achieve a timely response in case detection. It also suggests that asymptomatic cases can be identified at an early date if proper strategies were implemented. Such a finding is also in contrast with previous literature that argues that asymptomatic cases are hard to discern due to various personal health and environmental status [49]. As illustrated in Fig 5A, the distribution of the diagnosis interval of different cases varies. The mean duration of the imported cases from the date of arrival to the date of NAT tested positive is 2.71 days. However, it would take, on average, another 2.04 days for a lab confirmation, which suggests that China has taken a much strict approach for COVID-19 case prevention and control. In addition, the figure also reveals that the diagnosis interval varies substantially among different cases, which highlights that the strategy of centralized quarantine plus NAT may effectively identify the COVID-19 cases, which hence, restricted and minimized the spread size of COVID-19 among communities potentially.

### 4.2. The effects of quarantine and NAT

In early March of 2020, when the global pandemic was still in its initial outbreak, the JPC mechanism did not require inbound passengers to be centralized and quarantined at a designated facility. Instead, a 14-days home-quarantine or self-quarantine was allowed [50]. Despite the quarantine policy was later upgraded a few times through March of 2020, overall, the statistical results showed that these policies presented a significant effect on shrinking the diagnosis intervals of imported COVID-19 cases, as multi-setting of policies were introduced into the models.

Specifically, Table 1 shows that the early implementation of the home-quarantine policy and its upgraded policy with a target at the inbound travelers to Beijing (on March 11) and Shanghai (on March 13) did not have a significant effect on contracting the mean diagnosis interval. However, as the imported case continued to be detected on inbound international flights and in more cities, the quarantine policy was further updated by requiring all personnel to be quarantined at designated facilities (i.e., charter hotels contracted with local governments) for a 14-day of observation after arrival. Entrants were escorted and observed by medical staff to enable a timely response meanwhile to avoid any local exposure. The regional variation was found. For instance, while the diagnosis interval was not found to reduce after the implementation of these policies in Beijing and Shanghai, other regions, such as Yunnan (March 17) and Shaanxi provinces, were found to have increased by 1.7 days and Xi’an (March 17) by 0.74 days. We consider the outcomes could be interpreted for two reasons. First, it may attribute to the insufficient testing and lab capacity in most of the cities in the early stage of the imported case increase, which may prolong the process of case screening. Second, it may be because some cases may have quarantined more than once, which may also have prolonged the mean interval of confirmation. For instance, we found there were 11 cases that experienced quarantine twice, given that their first port of entries was not in Yunnan. As to Guangzhou, we did not find any significant effect of the centralized quarantine policy implemented in Guangzhou on March 19, though the sign of the margins reveal the trend was decreased.

These different outcomes suggest the complex distributional patterns of the imported COVID-19 cases among different regions and the pressing need for the expansion of local healthcare capacity for COVID-19 responses. It also reflects the adaptive strategies through a dynamic policy adjustment to achieve a swift risk control of imported COVID-19 cases.

In addition, models 10–13 examined the effect of the centralized quarantine and NAT implemented in different cities and regions. Specifically, no significant effect was found in Tianjin, where this joint policy was first implemented as a pilot on all inbound travelers (on March 21), or in Beijing, where the policy was formally enforced on all inbound travelers (on March 25). As the joint policy of centralized quarantine and NAT was implemented progressively and rapidly in more municipalities and provinces, such as Shanghai (March 26), Guangdong (March 27), Yunnan (March 27), Xi’an (March 28), and Heilongjiang (April 2), the results (Table 1) show that the diagnosis interval of imported COVID-19 case from its arrival confirmation was associated with the largest reduction (by 0.59–0.64 days) in Heilongjiang province. While prior to that, a positive effect was found in Shanghai, which suggests a prolonged interval (of 0.57–0.79 days) after the joint policy being adopted on March 26, a tendency of a slight reduction in intervals was also observed from 0.79 days to 0.57 days over the time. Overall, the key of the lagged effect in curbing the interval through implementing the quarantine and NAT strategies implies that the joint policies could have an impact as long as they were implemented in a coordinated manner over time. However, no expected effect would be received in the end without a wide adoption by other cities or regions.

### 4.3. The effects of flight service adjustment

Given the consideration that the risk of infection of the airborne pathogen, i.e., SARS and MERS, increases proportionally with a longer duration of exposure (e.g., >8h of flight) on air travel [51], meanwhile by assuming the average incubation period ranges from 5 to 6 days according to WHO [52], we summarized the distribution of the infection risk of COVID-19 during travel and the relationship between the mean interval of diagnosis and traveler’s self-reported health status in the sample. Fig 5B shows that the estimated median time of infection (between exposure to the virus and symptom onset), which ranges from 2 days (53 cases, or 5.9% of the sample, based on the assumption of a 14-day incubation period) to 5 days (395 cases, or 44% of the sample, based on the assumption of a 14-day incubation period) after the departure. Such a result is found similar to that released by WHO. Fig 5C provides a more detailed illustration of the distribution of time window of the SARS-CoV-2 virus infection associated with travel. The date of the potential infection was calculated using the date of confirmation minus the various incubation periods. Hence, the figure shows that quite a few cases were likely to be infected within two days, either prior to or after one’s departure date, which confirms a high risk of infection during travel.

However, given that little was known about SARS-CoV-2 virus at the early stage of the pandemic, to further reduce the risk of potential importation of COVID-19, the CAAC launched two corresponding service adjustments: the first strategy was the implementation of a progressive reduce the capacity of the inbound international flights in three stages [53]. The first stage was from January 23 to March 18, during which the volume of inbound international flights was reduced to 1,165 per week. In stage two (March 19–28), the volume was further reduced by 37% [54]. In stage three (started on March 29 and afterward) [55], the volume was decreased again through the implementation of the “five-one” policy, which required one airline of each country to retain only one international flight with one Chinese city per week [55]. The second strategy was to divert part of the international flights from risky epidemic countries (with a scheduled destination in Beijing) to other 12 Chinese cities to split the importation risk and the workload of screening for custom and border protection [56].

Our statistical analysis revealed that while the implementation of the second stage of service reduction has no effect on reducing the diagnosis interval if compared with the first stage (baseline). The third stage of flight reduction (through the implementation of the Five-One policy) demonstrated a strong association with the decline of diagnosis interval ranged from 0.36 days to 0.42 days compared with the variation observed in other stages. Such an effect attributes to two reasons. First, a continuous cancellation of inbound international flights, especially direct flight, has unavoidably suppressed the travel demand, especially from countries with a high risk of COVID-19 in March 2020 (such as Italy, Spain and U.K.). As a result, this indirectly promoted the surge of international passenger inflow through transfer via other cities and countries. This also explains the abrupt increased imported cases observed in Suifenhe, a border city of Heilongjiang, after implementing the second stage of flight service reduction, as a large number of passengers were found to enter the border through transiting either via or from Russia.

Such a phenomenon suggests that while the potential risk of inbound cases in major cities could be mitigated to some extent through a continuous reduction of international flight services, the risk could not be fundamentally eliminated since it might be simply shifted to other cities through an unusual detour. In addition, given that more stringent screening guidance was adopted by airline companies later in the pandemic [57], passengers with any clinical symptoms, such as cough, and high fever, were prohibited from boarding. These arrangements may also lead to a reduction of passengers, which hence, may have speeded up the efficiency of case screening and detection.

In addition, we also evaluated the effect of flight diversion with a focus on three major entry cities–Tianjin (implemented from March 21), Shanghai (March 23) and Xi’an (March 23). However, due to the data availability, our analysis was limited to only three out of the 12 cities that received diverted flights from Beijing. The results, as summarized in Table 1, reveals that the measure of diverting flights to alternative cities did not lessen the mean value of the diagnosis interval for Beijing. Additionally, cities that received inbound flight rerouted from Beijing showed a significantly prolonged diagnosis interval of 0.25–0.29 days since its implementation (as shown in Model 11 and 12). The results, though different from expectation, reflect the challenge brought by the large volume of inbound flights, passengers and potential imported COVID-19 risk both to Beijing and the alternate cities in terms of custom border scrutiny, case screening and medical treatment. For instance, it was reported that around 6,000–8,000 inbound passengers arrived in Beijing each day during the study period, nearly 3000 of them were later transferred for further medical treatment. Given such a challenging situation, it became imperative for the alternative cities to implement a more stringent border control and case screening with limited medical capacity and resources [58]. In fact, the arrangement that the alternative cities were selected to receive diverted flights from Beijing also de facto reveals the difficulty in shortening the diagnosis interval rapidly.

### 4.4. The effect of border closure strategy

On April 7, 2020, the containment of COVID-19 importation was characterized by a border closure of the Sino-Russia land port in Suifenhe of Heilongjiang province [59]. As illustrated in Fig 5D, this intervention was also in response to the upsurge of cases from Russia that emerged in late March due to modal transfer and detour. One salient feature of the cases identified in Suifenhe city was that the proportion of asymptomatic cases was much higher (188 off 533 cases, or 35.4%) than that in other cities. Due to the asymptomatic group had a significantly longer duration before presenting the symptom compared with the symptomatic group [60], the detection of asymptomatic cases can be more challenging, and it could further prolong the mean diagnosis interval. In addition, Fig 5D also reveals that the mean of diagnosis time from entry to a case confirmation is negatively correlated with the overall pandemic risk (measured in the number of cases per million people) among countries. Such a result suggests passengers who came from countries with a more severe pandemic risk tend to experience a relatively shorter diagnosis interval, which suggests that passengers from these countries tend to present either a COVID-19 related symptom relatively faster or had a relatively higher risk of being infected. In particular, those cases from Russia were found the largest, appears to be a mix with cases from various countries.

Given that the asymptomatic group cannot be lifted from centralized quarantine until negative NATs are confirmed on two consecutive samples at least 24 hours apart [61], in addition, given a large number of passengers continued arrived at the border custom in Heilongjiang province, the border was closed temporarily to allow the city of Suifenhe to have time to respond otherwise would have been overwhelmed [62]. In such an event, the marginal effect, as revealed in Table 1 (model 14), shows that the border closure contributes to a reduction of 0.69 days of diagnosis interval, which confirms to be effective in mitigating the imported COVID-19 risk.

### 4.5. Other control factors

The estimates of other control factors were found in general, consistent across various models. In particular, we found that city with a higher administrative level is statistically significantly associated with a much shorter diagnosis interval of imported cases on average, by 2.11–3.13 days. Conversely, the city with a higher GDP per capita is associated with a longer diagnosis time of 0.57–0.85 days. Such findings suggest that the effectiveness of imported case screening and identification does vary spatially. While major municipalities, such as Beijing and Shanghai, have a relatively higher level of resources for effective risk control of imported cases, a higher economic performance also implies that these cities tend to receive a large number of international passengers, which is likely to be associated with a relatively higher pressure of COVID-19 screening, and thus prolonged the diagnosis interval.

In addition, the analyses also reveal that the diagnosis interval also varies significantly among cities with different types of border of entries. For instance, cities with both borders of entry via air and land were associated with 2.03–3.36 days longer diagnosis interval than that with only the entry via air. Similarly, cities with both borders of entry via air and harbor were found to be associated with 0.86–0.99 more days of diagnosis interval for the imported cases. Such a result confirms that cities with multi-types of the port of entry are likely to experience more significant uncertainties of importation risk of COVID-19.

The level of policy stringency was also found to have a significant impact on reducing the diagnosis period by 0.01 days, suggesting that a collaborative effort of implementing stringent containment policies is conducive to rapid screening of imported COVID-19 cases.

## 5. Conclusions

The synchronized effect of the JPC mechanism, achieved through the multi-dimensional strategies, was confirmed effective in curbing the imported cases and compressing the diagnosis interval of re-entering COVID-19 cases from arrival to confirmation. During the early surge of the pandemic from March to May 2020, the implementation of a centralized quarantine plus a NAT to all inbound traveler was found the most effective in terms of cutting down the diagnosis interval of confirmation, which can be up to 0.64 days, when well-coordinated efforts with all stringent interventions persisted over time. It is worth noting that even for those intervals that have been increased before a synchronized effect emerged, the intervals were in a decreasing trend. In particular, the policies of flight service reduction were found to be associated with a reduction of the mean intervals of diagnosis from arrival to lab confirmation by 0.44 days maximally. Meanwhile, we found the border closure was associated with a reduction of the diagnosis interval of imported cases by 0.69 days.

One should also note that the study has several limitations, which need to be addressed in future research endeavors. For instance, the results were derived based on the limited size of the sample and it only captured the early surge of the pandemic while concentrating on a few cities. Hence, future studies may expand both the temporal and spatial scales of the data by collecting more cases reported by wider regions to further validate the findings. In addition, our evaluation only covered 17 major NPI strategies in response to imported COVID-19 risk control. Given that the pandemic is still ongoing and new strategies and measures were adapted dynamically, future research may focus on developing predictive modeling systems based on various monitoring data to achieve a timely policy evaluation.

Overall, our empirical study provides important implications for effective risk control on imported COVID-19 cases. Specifically, collective response to the pandemic across geographical and administrative boundaries is essential to achieve a timely control of the imported risk of COVID-19 [63]. The networked governance in dealing with coronavirus crises may provide valuable insights for both China and other countries to improve the consistency, reliability and effectiveness of policies to address the pandemic crisis.

## Supporting information

S1 Appendix (DOCX)
