# Supplementary material for: The effect of the synchronized multi-dimensional policies on imported COVID-19 curtailment in China
Source: PLoS One. 2021 Jun 1;16(6):e0252224. doi: 10.1371/journal.pone.0252224 (PMC8168853; doi:10.1371/journal.pone.0252224)
Supplement: S1 Appendix — (DOCX) [file pone.0252224.s001.docx]

**S1 File**

**S1 Fig. Distribution of the dependent variable (n=1,181, missing value=133)**


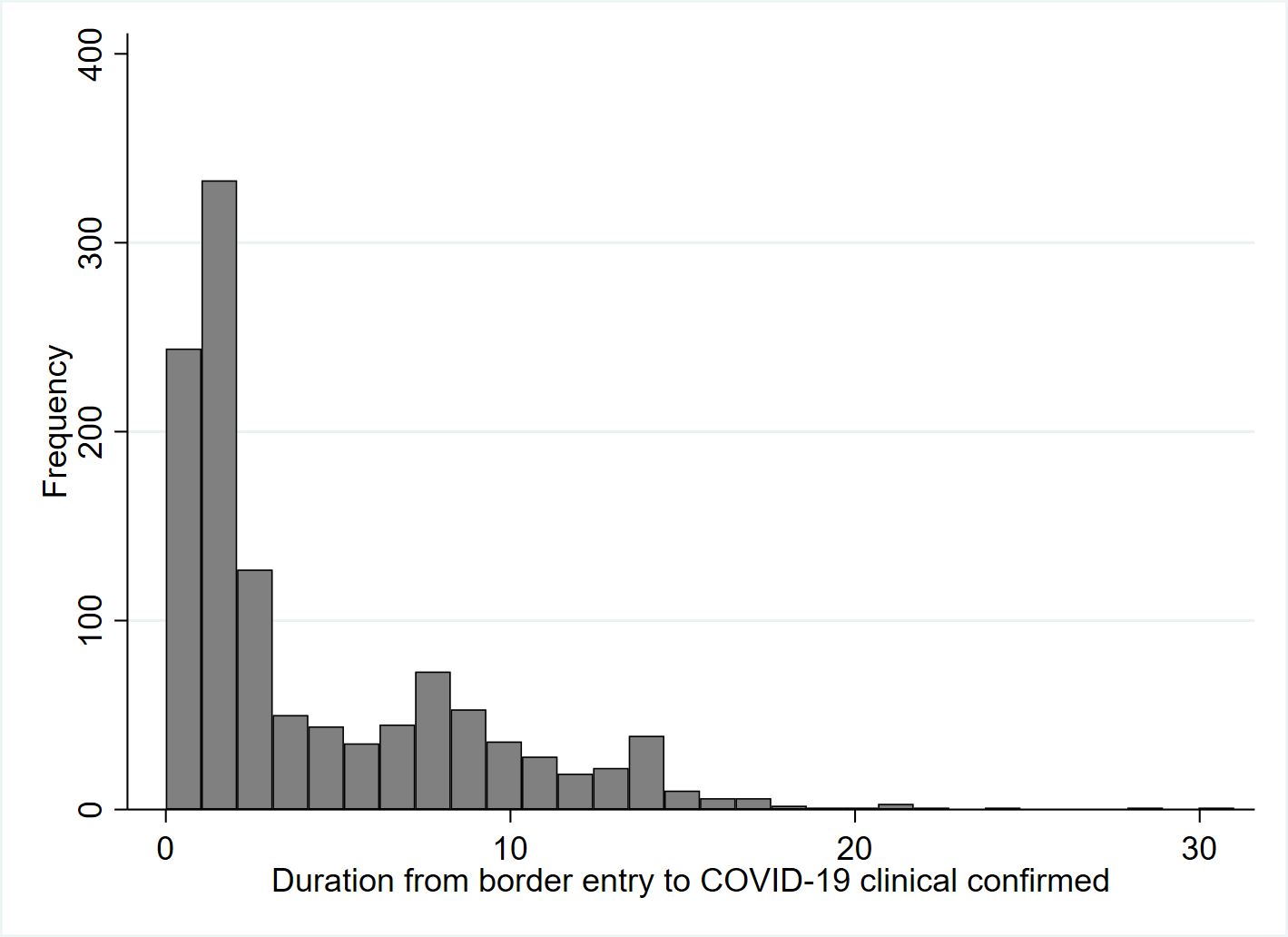


**S1 Table Policies in details.**

Table 1 description: Shortly after the confirmation of the first imported case on February 26, 2020 in Gansu province, on March 3, a 14-day quarantine policy for all oversea travelers was implemented in Beijing, with special attention to those from four countries with severe epidemics, including South Korea, Italy, Iran, and Japan. As the situation continued to deteriorate, a new quarantine policy that applied for a wider scale oversea travelers was implemented in Beijing (on March 11th) and in Shanghai (on March 13th). The new cases kept increasing from March 14 to 22. On March 16, oversea travelers entering Beijing were required to quarantine at a centralized observation point for 14 days. A similar policy was launched in Shanghai, Yuan and Shaanxi on March 17, and in Tianjin and Guangdong on March 18 and 19, respectively. At the same time, a total of 155 imported cases were confirmed from 11 countries. From March 18 to 22, the number of imported cases from the United Kingdom, Spain, France, the United States and the Philippines increased rapidly, which particularly increased the risk in Beijing. As a result, a new policy was implemented to divert certain inbound international flights to other 12 cities on March 23 and two days later, 14-day collective quarantine and mandatory testing were required for all international travelers arrived in Beijing. Similar policies were implemented in other cities and provinces, including Shanghai (on March 26), Guangdong and Yunnan (on March 27), Shaanxi (on March 28) and Heilongjiang (on April 2) afterward. On March 29, the "Five-One Policy", which allows one airline to serve one country, from one foreign city to one China, with no more than one flight per a week, was implemented by the China Civic Aviation Administration, to further cut international flights due to the concerns of the rising numbers of new imported cases. One direct consequence of this policy was that due to the cancellation of direct flights, oversea travelers had to reroute via Russia and enter China through the land port primarily in Heilongjiang province. On April 7, the major land port between Russia and China in Suifenghe was closed. Please note, all policies are coded according to each case’ date-of-entry.

**S1A Table Quarantine policy**

| No# | Date | Variable | Place | Policy Type | Details | Source |
| --- | --- | --- | --- | --- | --- | --- |
| 1 | 3 March 2020 | p303bjr | Beijing | Quarantine | On March 3, Beijing launched its first intervention policy against COVID-19 importation. Special quarantine policy was applied to those who arrived in Beijing via the Beijing International Airport from countries with severe epidemics, such as South Korea, Italy, Iran, and Japan. Those who has a residence in Beijing shall be included in the community prevention and control system and be quarantined at home for 14 days. Other travelers shall be arranged for a collective quarantine at a centralized medical observation in designated hotels for 14 days. | <https://www.sohu.com/a/377395134_255783> |
| 2 | 11 March 2020 | p311bjr | Beijing | Quarantine | Starting from March 11, all travelers who entered Beijing from non-severe epidemic countries must also be quarantined either at home or at a centralized medical observation in designated hotels for 14 days. | <https://www.sohu.com/a/379330699_200814> |
| 3 | 13 March2020 | p313shr | Shanghai | Quarantine | The Office of the Shanghai Leading Group for the Prevention and Control of COVID-19 announced on March 12: Starting from March 13, all Chinese and foreign personnel, who came from South Korea, Italy, Iran, Japan, France, Spain, Germany, and the United States, are all subject to home or centralized quarantine health observation for 14 days. | <http://sh.bendibao.com/news/2020313/218998.shtm> |
| 4 | 16 March 2020 | p316bjr | Beijing | Quarantine | Starting from March 16, all foreign personnel entering Beijing should be quarantined at a centralized observation facility for 14 days. The centralized observation point are equipped with professional medical care and staff, and routine health monitoring will be carried out. In a very rare and special circumstance, home observation may be permitted after a strict evaluation. | <http://bj.bendibao.com/news/2020316/271246.shtm> |
| 5 | 17 March 2020 | p317shr | Shanghai | Quarantine | Starting from March 17, all Chinese and foreign personnel who have had a history within 14 days before entering Shanghai, had traveled or a residence in 16 countries including South Korea, Italy, Iran, Japan, France, Spain, Germany, the United States, the United Kingdom, Switzerland, Sweden, Belgium, Norway, the Netherlands, Denmark, Austria, will be subject to home or centralized quarantine for 14 days. | <https://baijiahao.baidu.com/s?id=1660123348524886748&wfr=spider&for=pc> |
| 6 | 17 March 2020 | p317xar | Shaanxi province | Quarantine | On March 17, the Shaanxi Provincial Leading Group for the Response to the Epidemic issued the "Notice on Further Strengthening the Prevention and Control of the Epidemic of Persons Coming to and Returning to Shaanxi from Abroad", which implemented the most stringent management measures for inbound personnel to all travelers who came to and returned to Shaanxi from abroad. All travelers are required to be quarantined at a centralized medical observation facility for 14 days. |  |
| 7 | 17 March 2020 | p317ynr | Yunnan province | Quarantine | The Yunnan Provincial Leading Group for Response to the Epidemic issued a notice that starting from the March 17^th^, a centralized quarantine medical observation is required for those who have travelled abroad and have a destination in Yunnan within 14 days. |  |
| 8 | 18 March 2020 | p318tj | Tianjin | Quarantine | Starting from March 18, all persons entering Tianjin from any means should, in principle, be transferred to a centralized observation point for 14 days of centralized medical observation and quarantine. Those with symptoms, such as fever, shall be treated according to the prescribed procedures. | <https://m.gmw.cn/2020-03/17/content_1301058702.htm> |
| 9 | 19 March 2020 | p318gdr | Guangdong province | Quarantine | Starting from March 19, all travelers who arrived in Guangdong province from abroad, in particular, for those within 14 days before coming to Guangdong, stayed in South Korea, Italy, Iran, Japan, Germany, France, the United States, Spain, the United Kingdom, Thailand, the Philippines, Switzerland, Sweden, Belgium, Norway, the Netherlands, Denmark, Austria, Malaysia, must be quarantined at home or centralized isolation for medical observation for 14 days. | <https://xw.qq.com/cmsid/20200319A062Z300> |
| 10 | 21 March 2020 | p321tj | Tianjin | Quarantine+ Nucleic acid test | Starting from March 21, a centralized quarantine and nucleic acid test for COVID-19 are required for all travelers arrived in Tianjin. | <http://news.eastday.com/eastday/13news/auto/news/china/20200331/u7ai9189979.html> |
| 11 | 25 March 2020 | p325bj | Beijing | Quarantine+ Nucleic acid test | Starting from March 25, all travelers entered through the Port of Beijing, regardless of destination, will be quarantined and observed for 14 days. In addition, nucleic acid tests will be conducted. Those entering Beijing from other ports within 14 days will also be quarantined and undergo a nucleic acid testing. Travelers who arrived at other designated first entry point must be tested at the first entry point and will be quarantined for 14 days at a designated medical facility. | <http://bj.bendibao.com/news/2020324/271776.shtm> |
| 12 | 26 March 2020 | p326sh | Shanghai | Quarantine+ Nucleic acid test | Starting at 18:00 on March 26, all persons entering Shanghai will be subject to a 14-day quarantine health observation. In addition, the new coronavirus nucleic acid test will be implemented for all people entering Shanghai. All travelers who are in a centralized quarantine facility must pay for their accommodation and meals. | <https://www.takefoto.cn/viewnews-2094996.html> |
| 13 | 27 March 2020 | p327gd | Guangdong province | Quarantine+ Nucleic acid test | Starting at 18:00 on March 27th, all people entering the Guangdong port (including Hong Kong, Macao and Taiwan areas, including transit passengers) are required to take nucleic acid testing, and will be quarantined for 14 days for medical observation. All travelers who are in a centralized quarantine facility must pay for their accommodation and meals. | <https://www.takefoto.cn/viewnews-2094996.html> |
| 14 | 27 March 2020 | p327yn | Yunnan province | Quarantine+ Nucleic acid test | Starting from March 27, 2020, for those who have a history of overseas travel and enter Yunnan from an airport within 14 days (including transit personnel), both a centralized quarantine observation for 14 days and a nucleic acid testing are required. | <http://www.yn.gov.cn/ztgg/yqfk/zcfk/202003/t20200326_201199.html> |
| 15 | 28 March 2020 | p328xa | Shaanxi province | Quarantine+ Nucleic acid test | Starting from March 28, nucleic acid testing will be required for all persons entering the country through the ports of Shaanxi Province, and they will then be quarantined for 14 days. | <https://xw.qq.com/cmsid/20200328A0KLHX00> |
| 16 | 2 April, 2020 | p402hlj | Heilongjiang province | Quarantine+ Nucleic acid test | A strict quarantine and nucleic acid test policy was implemented on April 2. The policy requires a prevention and control of COVID-19 through six “100% - closed loop control” measures for all entry persons in the Heilongjiang province. Specifically, it requires a strict implementation of a 100% boarding and quarantine of inbound vehicles at all ports in the province, a 100% inspection of health declaration cards, a 100% inspection of all entry persons through body temperature testing, a 100% implementation of epidemiological investigation, a 100% sampling and testing, and a 100% implementation of centralized isolation. The specific management and control measures include "14 days of centralized quarantine medical observation + 14 days of home quarantine medical observation + 2 nucleic acid tests + 1 serum antibody (IgM and IgG) test" to ensure no case is missed. | <http://www.hlj.gov.cn/zwfb/system/2020/04/02/010923038.shtml> |

**S1B Table Flight service cut and re-routing measures.**

| 17 | 23 Jan-18 March 2020 | pfl-1 | All 7 cities and provinces | Flight service cut | From the beginning of the outbreak (on January 23) to March 19, the Civil Aviation Administration issued the first stage of the regulation known as the "Notice on Controlling International Shipping Passenger Traffic During the Period of Epidemic Prevention and Control". The maximum number of flights was limited to 1165 flights per week during the period. | <https://www.sohu.com/a/384275672_115362> |
| --- | --- | --- | --- | --- | --- | --- |
|  | 19-28 March 2020 | pfl-2 | All 7 cities and provinces | Flight service cut | The Civil Aviation Administration issued the second stage of regulation, which requires the total number of inbound international flights to be reduced to 734 during March 19 - 26, a decrease of 37% from the previous period. | <https://www.sohu.com/a/384275672_115362> |
|  | 21 March 2020 | pfr-2 | Tianjin | Receiving Re-routing flights from Beijing | Starting from March 21, Tianjin will undertake the diversion task of international flights destined for Beijing | <https://xw.qq.com/cmsid/20200331A0CXNC00> |
| 18 | 23 March 2020 | pfr-1 | Beijing | Re-routing flights | Starting from March 23, all international flights destined for Beijing will be diverted to the following 12 cities as the designated first entry points: Tianjin, Shijiazhuang, Taiyuan, Hohhot, Shanghai Pudong, Jinan, Qingdao, Nanjing, Shenyang, Dalian, Zhengzhou, and Xi'an. | <https://baijiahao.baidu.com/s?id=1661941716201836939&wfr=spider&for=pc> |
|  | 23 March 2020 | pfr-2 | Shanghai | Receiving diverted flights from Beijing | Same as above | <https://www.sohu.com/a/382133973_351146> |
|  | 23 March 2020 | pfr-2 | Xi’an | Receiving diverted flights from Beijing | Same as above | Same as above |
|  | 29 March, 2020 | pfl-3 | All 7 cities and provinces | Flight service cut | The third stage of flight regulation was implemented by the Civil Aviation Administration. The policy, which is known as “Five-One” policy, requires that each domestic airline can only retain one route to any country, and each route operates weekly. No more than one flight; each foreign airline company can only keep one route to China. | <http://cms2.newsduan.com/newsyun/zhuanti/2020/hrzxd/zysd/20200328/143222.html> |

**S1C Table Border closure**

| 19 | 7April 2020 | p407hlj | Heilongjiang province | Border closure | The China-Russian Suifenhe Port was officially closed from the 7th to the 13th, and has been closed since then. | <http://vladivostok.chineseconsulate.org/chn/zytz/t1768880.htm> |
| --- | --- | --- | --- | --- | --- | --- |

**S2 Table Descriptive statistics of all variables.**

| Variable | Variable description | Feature | Obs | Mean | Std. Dev. | Min | Max |
| --- | --- | --- | --- | --- | --- | --- | --- |
| v8n | Diagnosis interval | Count | 1181 | 4.75 | 4.37 | 0.00 | 31.00 |
| v2 | Transfer or not | Dummy | 1314 | 0.65 | 0.48 | 0.00 | 1.00 |
| v3 | Trip length (hr) | Continuous | 1312 | 12.61 | 3.71 | 1.57 | 31.25 |
| v4 | Administrative level | Categorical | 1314 | 1.84 | 0.37 | 1.00 | 2.00 |
| v5 | GDP per capita | Continuous | 1314 | 1.84 | 1.49 | 0.52 | 3.75 |
| v6 | Border type | Categorical | 1314 | 2.20 | 0.74 | 1.00 | 3.00 |
| v7 | Stringency level | Continuous | 1179 | 75.45 | 19.46 | 2.78 | 100.00 |
| v9 | Asymptomatic case | Dummy | 1314 | 0.17 | 0.37 | 0.00 | 1.00 |
| v12 | Symptomatic onset case upon arrival | Dummy | 1189 | 0.16 | 0.36 | 0.00 | 1.00 |
| v14 | No# of travelers associated with each confirmed case | Continuous | 1178 | 0.38 | 0.49 | 0.00 | 3.78 |
| v15 | Local daily new confirmed cases | Continuous | 1314 | 7.15 | 11.26 | 0.00 | 52.00 |
| ocountry1 | Angola | Dummy | 1314 | 0.00 | 0.04 | 0.00 | 1.00 |
| ocountry2 | Australia | Dummy | 1314 | 0.00 | 0.03 | 0.00 | 1.00 |
| ocountry3 | Austria | Dummy | 1314 | 0.00 | 0.05 | 0.00 | 1.00 |
| ocountry4 | Bangladesh | Dummy | 1314 | 0.00 | 0.05 | 0.00 | 1.00 |
| ocountry5 | Belgium | Dummy | 1314 | 0.00 | 0.05 | 0.00 | 1.00 |
| ocountry6 | Brazil | Dummy | 1314 | 0.01 | 0.07 | 0.00 | 1.00 |
| ocountry7 | Burkina Faso | Dummy | 1314 | 0.00 | 0.05 | 0.00 | 1.00 |
| ocountry8 | Cambodia | Dummy | 1314 | 0.00 | 0.06 | 0.00 | 1.00 |
| ocountry9 | Canada | Dummy | 1314 | 0.01 | 0.09 | 0.00 | 1.00 |
| ocountry10 | Chile | Dummy | 1314 | 0.00 | 0.03 | 0.00 | 1.00 |
| ocountry11 | Congo | Dummy | 1314 | 0.00 | 0.05 | 0.00 | 1.00 |
| ocountry12 | Cote d'Ivoire | Dummy | 1314 | 0.00 | 0.03 | 0.00 | 1.00 |
| ocountry13 | Ethiopia | Dummy | 1314 | 0.00 | 0.05 | 0.00 | 1.00 |
| ocountry14 | France | Dummy | 1314 | 0.04 | 0.20 | 0.00 | 1.00 |
| ocountry15 | Germany | Dummy | 1314 | 0.00 | 0.06 | 0.00 | 1.00 |
| ocountry16 | Greek | Dummy | 1314 | 0.00 | 0.03 | 0.00 | 1.00 |
| ocountry17 | Hungary | Dummy | 1314 | 0.00 | 0.06 | 0.00 | 1.00 |
| ocountry18 | Indonesia | Dummy | 1314 | 0.00 | 0.05 | 0.00 | 1.00 |
| ocountry19 | Iran | Dummy | 1314 | 0.01 | 0.07 | 0.00 | 1.00 |
| ocountry20 | Ireland | Dummy | 1314 | 0.00 | 0.05 | 0.00 | 1.00 |
| ocountry21 | Italy | Dummy | 1314 | 0.03 | 0.16 | 0.00 | 1.00 |
| ocountry22 | Japan | Dummy | 1314 | 0.00 | 0.06 | 0.00 | 1.00 |
| ocountry23 | Luxembourg | Dummy | 1314 | 0.00 | 0.03 | 0.00 | 1.00 |
| ocountry24 | Madagascar | Dummy | 1314 | 0.00 | 0.03 | 0.00 | 1.00 |
| ocountry25 | Malaysia | Dummy | 1314 | 0.00 | 0.06 | 0.00 | 1.00 |
| ocountry26 | Mexico | Dummy | 1314 | 0.00 | 0.04 | 0.00 | 1.00 |
| ocountry27 | Myanmar | Dummy | 1314 | 0.00 | 0.03 | 0.00 | 1.00 |
| ocountry28 | Netherland | Dummy | 1314 | 0.00 | 0.05 | 0.00 | 1.00 |
| ocountry29 | Niger | Dummy | 1314 | 0.00 | 0.03 | 0.00 | 1.00 |
| ocountry30 | Nigeria | Dummy | 1314 | 0.01 | 0.08 | 0.00 | 1.00 |
| ocountry31 | Norway | Dummy | 1314 | 0.00 | 0.03 | 0.00 | 1.00 |
| ocountry32 | Pakistan | Dummy | 1314 | 0.01 | 0.07 | 0.00 | 1.00 |
| ocountry33 | Philippines | Dummy | 1314 | 0.01 | 0.11 | 0.00 | 1.00 |
| ocountry34 | Portugal | Dummy | 1314 | 0.00 | 0.03 | 0.00 | 1.00 |
| ocountry35 | Russia | Dummy | 1314 | 0.52 | 0.50 | 0.00 | 1.00 |
| ocountry36 | Serbia | Dummy | 1314 | 0.00 | 0.06 | 0.00 | 1.00 |
| ocountry37 | Singapore | Dummy | 1314 | 0.00 | 0.05 | 0.00 | 1.00 |
| ocountry38 | Spain | Dummy | 1314 | 0.05 | 0.21 | 0.00 | 1.00 |
| ocountry39 | SriLanka | Dummy | 1314 | 0.00 | 0.03 | 0.00 | 1.00 |
| ocountry40 | Sweden | Dummy | 1314 | 0.00 | 0.05 | 0.00 | 1.00 |
| ocountry41 | Switzerland | Dummy | 1314 | 0.01 | 0.07 | 0.00 | 1.00 |
| ocountry42 | Taiwan | Dummy | 1314 | 0.00 | 0.03 | 0.00 | 1.00 |
| ocountry43 | Thailand | Dummy | 1314 | 0.01 | 0.08 | 0.00 | 1.00 |
| ocountry44 | Togolese | Dummy | 1314 | 0.00 | 0.03 | 0.00 | 1.00 |
| ocountry45 | Turkey | Dummy | 1314 | 0.00 | 0.03 | 0.00 | 1.00 |
| ocountry46 | UAE | Dummy | 1314 | 0.01 | 0.08 | 0.00 | 1.00 |
| ocountry47 | UK | Dummy | 1314 | 0.15 | 0.36 | 0.00 | 1.00 |
| ocountry48 | USA | Dummy | 1314 | 0.09 | 0.28 | 0.00 | 1.00 |
| tourist | Travel as student | Dummy | 444 | 0.12 | 0.33 | 0.00 | 1.00 |
| student | Travel as student | Dummy | 444 | 0.50 | 0.50 | 0.00 | 1.00 |
| resident | Residential-related travel | Dummy | 443 | 0.09 | 0.29 | 0.00 | 1.00 |
| job | Working-related travel | Dummy | 444 | 0.29 | 0.46 | 0.00 | 1.00 |
| p311bjr | Quarantine policy, Beijing | Dummy | 1314 | 0.00 | 0.06 | 0.00 | 1.00 |
| p313shr | Quarantine policy, Shanghai | Dummy | 1314 | 0.00 | 0.06 | 0.00 | 1.00 |
| p316bjr | Upgraded quarantine policy, Beijing | Dummy | 1314 | 0.01 | 0.11 | 0.00 | 1.00 |
| p317shr | Updated quarantine policy, Shanghai | Dummy | 1314 | 0.09 | 0.28 | 0.00 | 1.00 |
| p317ynr | Updated quarantine policy, Yunnan | Dummy | 1314 | 0.01 | 0.08 | 0.00 | 1.00 |
| p317xar | Updated quarantine policy, Xi'an | Dummy | 1314 | 0.01 | 0.07 | 0.00 | 1.00 |
| p319gdr | Quarantine policy, Guangdong | Dummy | 1314 | 0.02 | 0.15 | 0.00 | 1.00 |
| p321tj | Quarantine & Testing, Tianjin | Dummy | 1314 | 0.05 | 0.22 | 0.00 | 1.00 |
| p325bj | Quarantine & Testing, Beijing | Dummy | 1314 | 0.10 | 0.30 | 0.00 | 1.00 |
| p326sh | Quarantine & Testing, Shanghai | Dummy | 1314 | 0.12 | 0.32 | 0.00 | 1.00 |
| p327yn | Quarantine & Testing, Yunnan | Dummy | 1314 | 0.00 | 0.03 | 0.00 | 1.00 |
| p327gd | Quarantine & Testing, Guangdong | Dummy | 1314 | 0.04 | 0.19 | 0.00 | 1.00 |
| p328xa | Quarantine & Testing, Xi'an | Dummy | 1314 | 0.05 | 0.22 | 0.00 | 1.00 |
| p402hlj | Quarantine & Testing, Heilongjiang | Dummy | 1314 | 0.36 | 0.48 | 0.00 | 1.00 |
| p407hlj | Border closure, Heilongjiang | Dummy | 1314 | 0.03 | 0.16 | 0.00 | 1.00 |
| pfl | Flight reduction | Categorical | 1314 | 2.63 | 0.63 | 1.00 | 3.00 |
| pfr | Flight diversion | Categorical | 1314 | 0.62 | 0.87 | 0.00 | 2.00 |

**S3 Table Comparison of the conditional variance and conditional mean of policy variable.**

| Policy | Level | Conditional mean | Conditional variance | Count | Conditional variance > mean |
| --- | --- | --- | --- | --- | --- |
| p311bjr | 0 | 4.73 | 19.03 | 1177 | 14.30 |
|  | 1 | 9.75 | 28.25 | 4 | 18.50 |
|  | Total | 4.75 | 19.12 | 1181 | 14.37 |
| p313shr | 0 | 4.74 | 19.14 | 1176 | 14.40 |
|  | 1 | 5.60 | 16.30 | 5 | 10.70 |
|  | Total | 4.75 | 19.12 | 1181 | 14.37 |
| p316bjr | 0 | 4.73 | 18.92 | 1166 | 14.19 |
|  | 1 | 6.00 | 35.14 | 15 | 29.14 |
|  | Total | 4.75 | 19.12 | 1181 | 14.37 |
| p317shr | 0 | 4.87 | 19.96 | 1065 | 15.09 |
|  | 1 | 3.64 | 10.14 | 116 | 6.50 |
|  | Total | 4.75 | 19.12 | 1181 | 14.37 |
| p317ynr | 0 | 4.71 | 19.01 | 1173 | 14.30 |
|  | 1 | 10.00 | 8.86 | 8 | -1.14 |
|  | Total | 4.75 | 19.12 | 1181 | 14.37 |
| p317xar | 0 | 4.75 | 19.11 | 1174 | 14.36 |
|  | 1 | 4.14 | 23.14 | 7 | 19.00 |
|  | Total | 4.75 | 19.12 | 1181 | 14.37 |
| p319gdr | 0 | 4.79 | 19.26 | 1152 | 14.47 |
|  | 1 | 3.14 | 11.34 | 29 | 8.20 |
|  | Total | 4.75 | 19.12 | 1181 | 14.37 |
| p321tj | 0 | 4.85 | 19.00 | 1116 | 14.15 |
|  | 1 | 2.92 | 17.88 | 65 | 14.96 |
|  | Total | 4.75 | 19.12 | 1181 | 14.37 |
| p325bj | 0 | 4.75 | 19.13 | 1180 | 14.38 |
|  | 1 | 3.00 | . | 1 | -3.00 |
|  | Total | 4.75 | 19.12 | 1181 | 14.37 |
| p326sh | 0 | 5.02 | 20.13 | 1024 | 15.11 |
|  | 1 | 2.96 | 8.95 | 157 | 5.99 |
|  | Total | 4.75 | 19.12 | 1181 | 14.37 |
| p327yn | 0 | 4.75 | 19.13 | 1180 | 14.38 |
|  | 1 | 1.00 | . | 1 | -1.00 |
|  | Total | 4.75 | 19.12 | 1181 | 14.37 |
| p327gd | 0 | 4.84 | 19.54 | 1130 | 14.70 |
|  | 1 | 2.71 | 5.49 | 51 | 2.78 |
|  | Total | 4.75 | 19.12 | 1181 | 14.37 |
| p328xa | 0 | 4.94 | 19.54 | 1112 | 14.60 |
|  | 1 | 1.65 | 2.23 | 69 | 0.58 |
|  | Total | 4.75 | 19.12 | 1181 | 14.37 |
| p402hlj | 0 | 4.25 | 20.44 | 714 | 16.19 |
|  | 1 | 5.51 | 16.19 | 467 | 10.68 |
|  | Total | 4.75 | 19.12 | 1181 | 14.37 |
| p407hlj | 0 | 4.80 | 19.37 | 1149 | 14.57 |
|  | 1 | 2.72 | 6.27 | 32 | 3.55 |
|  | Total | 4.75 | 19.12 | 1181 | 14.37 |
| pfl | 1 | 5.53 | 30.37 | 105 | 24.84 |
|  | 2 | 4.25 | 18.54 | 281 | 14.29 |
|  | 3 | 4.82 | 17.73 | 795 | 12.91 |
|  | Total | 4.75 | 19.12 | 1181 | 14.37 |
| pfr | 0 | 5.56 | 20.41 | 834 | 14.85 |
|  | 1 | 5.50 | 58.30 | 6 | 52.80 |
|  | 2 | 2.73 | 9.80 | 341 | 7.07 |
|  | Total | 4.75 | 19.12 | 1181 | 14.37 |

Note: The table above shows the average numbers of diagnosis days affected by the implementation of different policies, which suggest that policy type is an estimator for predicting the number of diagnosis days shortened or prolonged. It is because the mean value of the outcome appears to vary by policy that implemented, the variances within each level of policy are higher than the means within each level. Hence, these differences suggest that over-dispersion is present and that a Negative Binomial model would be appropriate.

**S4A Table Pearson correlation table of policy variables**

|  | p311bjr | p313shr | p316bjr | p317shr | p317ynr | p317xar | p319gdr | p321tj | p325bj | p326sh | p327yn | p327gd | p328xa | p402hlj | p407hlj | pfl | pfr |
| --- | --- | --- | --- | --- | --- | --- | --- | --- | --- | --- | --- | --- | --- | --- | --- | --- | --- |
| p311bjr | 1 |  |  |  |  |  |  |  |  |  |  |  |  |  |  |  |  |
|  |  |  |  |  |  |  |  |  |  |  |  |  |  |  |  |  |  |
| p313shr | 0.00 | 1 |  |  |  |  |  |  |  |  |  |  |  |  |  |  |  |
|  | 0.90 |  |  |  |  |  |  |  |  |  |  |  |  |  |  |  |  |
|  |  |  |  |  |  |  |  |  |  |  |  |  |  |  |  |  |  |
| p316bjr | -0.01 | -0.01 | 1 |  |  |  |  |  |  |  |  |  |  |  |  |  |  |
|  | 0.83 | 0.81 |  |  |  |  |  |  |  |  |  |  |  |  |  |  |  |
|  |  |  |  |  |  |  |  |  |  |  |  |  |  |  |  |  |  |
| p317shr | -0.02 | -0.02 | -0.03 | 1 |  |  |  |  |  |  |  |  |  |  |  |  |  |
|  | 0.53 | 0.49 | 0.23 |  |  |  |  |  |  |  |  |  |  |  |  |  |  |
|  |  |  |  |  |  |  |  |  |  |  |  |  |  |  |  |  |  |
| p317ynr | 0.00 | 0.00 | -0.01 | -0.02 | 1 |  |  |  |  |  |  |  |  |  |  |  |  |
|  | 0.88 | 0.86 | 0.76 | 0.38 |  |  |  |  |  |  |  |  |  |  |  |  |  |
|  |  |  |  |  |  |  |  |  |  |  |  |  |  |  |  |  |  |
| p317xar | 0.00 | 0.00 | -0.01 | -0.02 | -0.01 | 1 |  |  |  |  |  |  |  |  |  |  |  |
|  | 0.88 | 0.87 | 0.78 | 0.41 | 0.84 |  |  |  |  |  |  |  |  |  |  |  |  |
|  |  |  |  |  |  |  |  |  |  |  |  |  |  |  |  |  |  |
| p319gdr | -0.01 | -0.01 | -0.02 | -0.05 | -0.01 | -0.01 | 1 |  |  |  |  |  |  |  |  |  |  |
|  | 0.76 | 0.74 | 0.56 | 0.09 | 0.67 | 0.69 |  |  |  |  |  |  |  |  |  |  |  |
|  |  |  |  |  |  |  |  |  |  |  |  |  |  |  |  |  |  |
| p321tj | -0.01 | -0.01 | -0.02 | -0.07* | -0.02 | -0.02 | -0.03 | 1 |  |  |  |  |  |  |  |  |  |
|  | 0.65 | 0.61 | 0.37 | 0.01 | 0.52 | 0.55 | 0.21 |  |  |  |  |  |  |  |  |  |  |
|  |  |  |  |  |  |  |  |  |  |  |  |  |  |  |  |  |  |
| p325bj | -0.02 | -0.02 | -0.04 | -0.10* | -0.03 | -0.02 | -0.05 | -0.08* | 1 |  |  |  |  |  |  |  |  |
|  | 0.50 | 0.45 | 0.19 | 0.00 | 0.34 | 0.37 | 0.07 | 0.01 |  |  |  |  |  |  |  |  |  |
|  |  |  |  |  |  |  |  |  |  |  |  |  |  |  |  |  |  |
| p326sh | -0.02 | -0.02 | -0.04 | -0.11* | -0.03 | -0.03 | -0.06* | -0.08* | -0.12* | 1 |  |  |  |  |  |  |  |
|  | 0.46 | 0.41 | 0.15 | 0.00 | 0.30 | 0.33 | 0.04 | 0.00 | 0.00 |  |  |  |  |  |  |  |  |
|  |  |  |  |  |  |  |  |  |  |  |  |  |  |  |  |  |  |
| p327yn | 0.00 | 0.00 | 0.00 | -0.01 | 0.00 | 0.00 | 0.00 | -0.01 | -0.01 | -0.01 | 1 |  |  |  |  |  |  |
|  | 0.96 | 0.95 | 0.91 | 0.76 | 0.94 | 0.94 | 0.88 | 0.82 | 0.74 | 0.71 |  |  |  |  |  |  |  |
|  |  |  |  |  |  |  |  |  |  |  |  |  |  |  |  |  |  |
| p327gd | -0.01 | -0.01 | -0.02 | -0.06* | -0.02 | -0.01 | -0.03 | -0.05 | -0.10* | -0.07* | -0.01 | 1 |  |  |  |  |  |
|  | 0.69 | 0.65 | 0.43 | 0.02 | 0.57 | 0.59 | 0.27 | 0.10 | 0.01 | 0.01 | 0.84 |  |  |  |  |  |  |
|  |  |  |  |  |  |  |  |  |  |  |  |  |  |  |  |  |  |
| p328xa | -0.01 | -0.01 | -0.03 | -0.07* | -0.02 | -0.02 | -0.04 | -0.05 | -0.08* | -0.09* | -0.01 | -0.05 | 1 |  |  |  |  |
|  | 0.64 | 0.60 | 0.36 | 0.01 | 0.50 | 0.53 | 0.20 | 0.05 | 0.00 | 0.00 | 0.81 | 0.09 |  |  |  |  |  |
|  |  |  |  |  |  |  |  |  |  |  |  |  |  |  |  |  |  |
| p402hlj | -0.04 | -0.05 | -0.08* | -0.23* | -0.06* | -0.05* | -0.11* | -0.17* | -0.25* | -0.27* | -0.02 | -0.15* | -0.18* | 1 |  |  |  |
|  | 0.14 | 0.10 | 0.00 | 0.00 | 0.03 | 0.05 | 0.00 | 0.00 | 0.00 | 0.00 | 0.46 | 0.00 | 0.00 |  |  |  |  |
|  |  |  |  |  |  |  |  |  |  |  |  |  |  |  |  |  |  |
| p407hlj | -0.01 | -0.01 | -0.02 | -0.05 | -0.01 | -0.01 | -0.02 | -0.04 | -0.05 | -0.06* | 0.00 | -0.03 | -0.04 | 0.22* | 1 |  |  |
|  | 0.75 | 0.72 | 0.53 | 0.07 | 0.65 | 0.67 | 0.38 | 0.18 | 0.05 | 0.03 | 0.87 | 0.24 | 0.17 | 0.00 |  |  |  |
|  |  |  |  |  |  |  |  |  |  |  |  |  |  |  |  |  |  |
| pfl | -0.14* | -0.16* | -0.12* | -0.39* | -0.16* | -0.14* | -0.15* | -0.09* | 0.20* | 0.14* | 0.02 | 0.04 | 0.14* | 0.44* | 0.10* | 1 |  |
|  | 0.00 | 0.00 | 0.00 | 0.00 | 0.00 | 0.00 | 0.00 | 0.00 | 0.00 | 0.00 | 0.55 | 0.17 | 0.00 | 0.00 | 0.00 |  |  |
|  |  |  |  |  |  |  |  |  |  |  |  |  |  |  |  |  |  |
| pfr | -0.04 | -0.04 | -0.04 | -0.01 | -0.06* | 0.00 | -0.11* | 0.36* | 0.15* | 0.58* | -0.02 | -0.14* | 0.37* | -0.53* | -0.12* | 0.11* | 1 |
|  | 0.15 | 0.11 | 0.11 | 0.79 | 0.04 | 0.87 | 0.00 | 0.00 | 0.00 | 0.00 | 0.47 | 0.00 | 0.00 | 0.00 | 0.00 | 0.00 |  |

Note: 1, The output based on 1314 obviations contains: (1) the Pearson correlation coefficient "*r*" is shown in the first row; and (2) the second row shows the significant level of p-value (p<=.05) which labeled with start. 2, We follow the critical value of general guidelines that provided by Cohen (1988) [SI.1] on | r | > .5 as large/strong correlation and highlight in yellow for negative correlation and green for positive correlation. 3, Correlation explanation: the "Quarantine+Testing" was upgraded in Shanghai since March 26th, 2020 ("p326sh") as the re-routing of inbound flight ("pfr") from Beijing to other cities was launched. Meanwhile, the re-routed inbound flights negatively affected the implementation of "Quarantine+Testing" in Heilongjiang ("p402hlj").

|  |
| --- |

**S4B Table** **Pearson correlation table of key control variables.**

|  |  | v2 | v3 | v4 | v5_1 | v6 | v7 | v9 |
| --- | --- | --- | --- | --- | --- | --- | --- | --- |
| v2 |  | 1 |  |  |  |  |  |  |
| v3 |  | 0.64* | 1 |  |  |  |  |  |
| v4 |  | 0.22* | 0.24* | 1 |  |  |  |  |
| v5_1 |  | -0.43* | -0.14* | 0.29* | 1 |  |  |  |
| v6 |  | -0.01 | 0.08* | -0.04 | 0.10* | 1 |  |  |
| v7 |  | -0.06 | -0.12* | 0.20* | -0.31* | -0.29* | 1 |  |
| v9 |  | 0.20* | 0.09* | 0.09* | -0.35* | -0.12* | 0.21* | 1 |
| v12 |  | -0.39* | -0.26* | -0.05 | 0.35* | 0.23* | 0.02 | -0.21* |
| v14 |  | 0.00 | 0.04 | -0.09 | 0.44* | 0.28* | -0.46* | -0.19* |
| v15 |  | -0.33* | -0.20* | 0.15* | 0.54* | 0.18* | -0.05 | -0.25* |
| resident |  | -0.09 | -0.02 | 0.08 | 0.09 | 0.02 | 0.08 | -0.02 |
| student |  | 0.11* | 0.33* | 0.13* | 0.14* | -0.16* | 0.02 | 0.06 |
| tourist |  | -0.02 | -0.10* | 0.00 | -0.05 | 0.04 | -0.05 | 0.00 |
| job |  | -0.06 | -0.28* | -0.20 | -0.16* | 0.10* | -0.05 | -0.08 |
|  |  |  |  |  |  |  |  |  |
|  |  | v12 | v14 | v15 | resident | student | tourist | job |
| v12 |  | 1 |  |  |  |  |  |  |
| v14 |  | 0.09* | 1 |  |  |  |  |  |
| v15 |  | 0.50* | 0.14* | 1 |  |  |  |  |
| resident |  | 0.09 | -0.06 | 0.08 | 1 |  |  |  |
| student |  | 0.06 | 0.05 | 0.15* | -0.07 | 1 |  |  |
| tourist |  | -0.13* | 0.10* | -0.05 | -0.02 | -0.37* | 1 |  |
| job |  | -0.03 | -0.08 | -0.18 | -0.16* | -0.63* | -0.24* | 1 |

Note:1, The output based on 1314 obviations contains: (1) the Pearson correlation coefficient "r" is shown in the first row, and (2) start shows significant level of p-value (p<=.05). 2, We follow the critical value of general guidelines that provided by Cohen (1988) [SI.1] on | r | > .5 as large/strong correlation and highlight in yellow for negative correlation and green for positive correlation. 3, Correlation explanation: the number of local daily new confirmed case increased as the imported confirmed cases rising from relatively higher GDP per capita countries.

|  |
| --- |
|  |
|  |
|  |
|  |

**S4C Table Pearson correlation table of original country variables.**

|  | ocountry1 | ocountry2 | ocountry3 | ocountry4 | ocountry5 | ocountry6 | ocountry7 | ocountry8 | ocountry9 | ocountry10 | ocountry11 | ocountry12 | ocountry13 | ocountry14 | ocountry15 | ocountry16 |
| --- | --- | --- | --- | --- | --- | --- | --- | --- | --- | --- | --- | --- | --- | --- | --- | --- |
| ocountry1 | 1 |  |  |  |  |  |  |  |  |  |  |  |  |  |  |  |
| ocountry2 | 0.00 | 1.00 |  |  |  |  |  |  |  |  |  |  |  |  |  |  |
| ocountry3 | 0.00 | 0.00 | 1.00 |  |  |  |  |  |  |  |  |  |  |  |  |  |
| ocountry4 | 0.00 | 0.00 | 0.00 | 1.00 |  |  |  |  |  |  |  |  |  |  |  |  |
| ocountry5 | 0.00 | 0.00 | 0.00 | 0.00 | 1.00 |  |  |  |  |  |  |  |  |  |  |  |
| ocountry6 | 0.00 | 0.00 | 0.00 | 0.00 | 0.00 | 1.00 |  |  |  |  |  |  |  |  |  |  |
| ocountry7 | 0.00 | 0.00 | 0.00 | 0.00 | 0.00 | 0.00 | 1.00 |  |  |  |  |  |  |  |  |  |
| ocountry8 | 0.00 | 0.00 | 0.00 | 0.00 | 0.00 | 0.00 | 0.00 | 1.00 |  |  |  |  |  |  |  |  |
| ocountry9 | 0.00 | 0.00 | 0.00 | 0.00 | 0.00 | -0.01 | 0.00 | -0.01 | 1.00 |  |  |  |  |  |  |  |
| ocountry10 | 0.00 | 0.00 | 0.00 | 0.00 | 0.00 | 0.00 | 0.00 | 0.00 | 0.00 | 1.00 |  |  |  |  |  |  |
| ocountry11 | 0.00 | 0.00 | 0.00 | 0.00 | 0.00 | 0.00 | 0.00 | 0.00 | 0.00 | 0.00 | 1.00 |  |  |  |  |  |
| ocountry12 | 0.00 | 0.00 | 0.00 | 0.00 | 0.00 | 0.00 | 0.00 | 0.00 | 0.00 | 0.00 | 0.00 | 1.00 |  |  |  |  |
| ocountry13 | 0.00 | 0.00 | 0.00 | 0.00 | 0.00 | 0.00 | 0.00 | 0.00 | 0.00 | 0.00 | 0.00 | 0.00 | 1.00 |  |  |  |
| ocountry14 | -0.01 | -0.01 | -0.01 | -0.01 | -0.01 | -0.01 | -0.01 | -0.01 | -0.02 | -0.01 | -0.01 | -0.01 | -0.01 | 1.00 |  |  |
| ocountry15 | 0.00 | 0.00 | 0.00 | 0.00 | 0.00 | 0.00 | 0.00 | 0.00 | -0.01 | 0.00 | 0.00 | 0.00 | 0.00 | -0.01 | 1.00 |  |
| ocountry16 | 0.00 | 0.00 | 0.00 | 0.00 | 0.00 | 0.00 | 0.00 | 0.00 | 0.00 | 0.00 | 0.00 | 0.00 | 0.00 | -0.01 | 0.00 | 1.00 |
|  | ocountry1 | ocountry2 | ocountry3 | ocountry4 | ocountry5 | ocountry6 | ocountry7 | ocountry8 | ocountry9 | ocountry10 | ocountry11 | ocountry12 | ocountry13 | ocountry14 | ocountry15 | ocountry16 |
| ocountry17 | 0.00 | 0.00 | 0.00 | 0.00 | 0.00 | 0.00 | 0.00 | 0.00 | -0.01 | 0.00 | 0.00 | 0.00 | 0.00 | -0.01 | 0.00 | 0.00 |
| ocountry18 | 0.00 | 0.00 | 0.00 | 0.00 | 0.00 | 0.00 | 0.00 | 0.00 | 0.00 | 0.00 | 0.00 | 0.00 | 0.00 | -0.01 | 0.00 | 0.00 |
| ocountry19 | 0.00 | 0.00 | 0.00 | 0.00 | 0.00 | 0.00 | 0.00 | 0.00 | -0.01 | 0.00 | 0.00 | 0.00 | 0.00 | -0.01 | 0.00 | 0.00 |
| ocountry20 | 0.00 | 0.00 | 0.00 | 0.00 | 0.00 | 0.00 | 0.00 | 0.00 | 0.00 | 0.00 | 0.00 | 0.00 | 0.00 | -0.01 | 0.00 | 0.00 |
| ocountry21 | -0.01 | 0.00 | -0.01 | -0.01 | -0.01 | -0.01 | -0.01 | -0.01 | -0.02 | 0.00 | -0.01 | 0.00 | -0.01 | -0.04 | -0.01 | 0.00 |
| ocountry22 | 0.00 | 0.00 | 0.00 | 0.00 | 0.00 | 0.00 | 0.00 | 0.00 | -0.01 | 0.00 | 0.00 | 0.00 | 0.00 | -0.01 | 0.00 | 0.00 |
| ocountry23 | 0.00 | 0.00 | 0.00 | 0.00 | 0.00 | 0.00 | 0.00 | 0.00 | 0.00 | 0.00 | 0.00 | 0.00 | 0.00 | -0.01 | 0.00 | 0.00 |
| ocountry24 | 0.00 | 0.00 | 0.00 | 0.00 | 0.00 | 0.00 | 0.00 | 0.00 | 0.00 | 0.00 | 0.00 | 0.00 | 0.00 | -0.01 | 0.00 | 0.00 |
| ocountry25 | 0.00 | 0.00 | 0.00 | 0.00 | 0.00 | 0.00 | 0.00 | 0.00 | -0.01 | 0.00 | 0.00 | 0.00 | 0.00 | -0.01 | 0.00 | 0.00 |
| ocountry26 | 0.00 | 0.00 | 0.00 | 0.00 | 0.00 | 0.00 | 0.00 | 0.00 | 0.00 | 0.00 | 0.00 | 0.00 | 0.00 | -0.01 | 0.00 | 0.00 |
| ocountry27 | 0.00 | 0.00 | 0.00 | 0.00 | 0.00 | 0.00 | 0.00 | 0.00 | 0.00 | 0.00 | 0.00 | 0.00 | 0.00 | -0.01 | 0.00 | 0.00 |
| ocountry28 | 0.00 | 0.00 | 0.00 | 0.00 | 0.00 | 0.00 | 0.00 | 0.00 | 0.00 | 0.00 | 0.00 | 0.00 | 0.00 | -0.01 | 0.00 | 0.00 |
| ocountry29 | 0.00 | 0.00 | 0.00 | 0.00 | 0.00 | 0.00 | 0.00 | 0.00 | 0.00 | 0.00 | 0.00 | 0.00 | 0.00 | -0.01 | 0.00 | 0.00 |
| ocountry30 | 0.00 | 0.00 | 0.00 | 0.00 | 0.00 | -0.01 | 0.00 | 0.00 | -0.01 | 0.00 | 0.00 | 0.00 | 0.00 | -0.02 | 0.00 | 0.00 |
| ocountry31 | 0.00 | 0.00 | 0.00 | 0.00 | 0.00 | 0.00 | 0.00 | 0.00 | 0.00 | 0.00 | 0.00 | 0.00 | 0.00 | -0.01 | 0.00 | 0.00 |
| ocountry32 | 0.00 | 0.00 | 0.00 | 0.00 | 0.00 | -0.01 | 0.00 | 0.00 | -0.01 | 0.00 | 0.00 | 0.00 | 0.00 | -0.02 | 0.00 | 0.00 |
|  | ocountry1 | ocountry2 | ocountry3 | ocountry4 | ocountry5 | ocountry6 | ocountry7 | ocountry8 | ocountry9 | ocountry10 | ocountry11 | ocountry12 | ocountry13 | ocountry14 | ocountry15 | ocountry16 |
| ocountry33 | 0.00 | 0.00 | -0.01 | -0.01 | -0.01 | -0.01 | -0.01 | -0.01 | -0.01 | 0.00 | -0.01 | 0.00 | -0.01 | -0.02 | -0.01 | 0.00 |
| ocountry34 | 0.00 | 0.00 | 0.00 | 0.00 | 0.00 | 0.00 | 0.00 | 0.00 | 0.00 | 0.00 | 0.00 | 0.00 | 0.00 | -0.01 | 0.00 | 0.00 |
| ocountry35 | -0.04 | -0.03 | -0.05 | -0.05 | -0.05 | -0.07 | -0.05 | -0.06 | -0.10 | -0.03 | -0.05 | -0.03 | -0.05 | -0.22 | -0.06 | -0.03 |
| ocountry36 | 0.00 | 0.00 | 0.00 | 0.00 | 0.00 | 0.00 | 0.00 | 0.00 | -0.01 | 0.00 | 0.00 | 0.00 | 0.00 | -0.01 | 0.00 | 0.00 |
| ocountry37 | 0.00 | 0.00 | 0.00 | 0.00 | 0.00 | 0.00 | 0.00 | 0.00 | 0.00 | 0.00 | 0.00 | 0.00 | 0.00 | -0.01 | 0.00 | 0.00 |
| ocountry38 | -0.01 | -0.01 | -0.01 | -0.01 | -0.01 | -0.01 | -0.01 | -0.01 | -0.02 | -0.01 | -0.01 | -0.01 | -0.01 | -0.05 | -0.01 | -0.01 |
| ocountry39 | 0.00 | 0.00 | 0.00 | 0.00 | 0.00 | 0.00 | 0.00 | 0.00 | 0.00 | 0.00 | 0.00 | 0.00 | 0.00 | -0.01 | 0.00 | 0.00 |
| ocountry40 | 0.00 | 0.00 | 0.00 | 0.00 | 0.00 | 0.00 | 0.00 | 0.00 | 0.00 | 0.00 | 0.00 | 0.00 | 0.00 | -0.01 | 0.00 | 0.00 |
| ocountry41 | 0.00 | 0.00 | 0.00 | 0.00 | 0.00 | -0.01 | 0.00 | 0.00 | -0.01 | 0.00 | 0.00 | 0.00 | 0.00 | -0.02 | 0.00 | 0.00 |
| ocountry42 | 0.00 | 0.00 | 0.00 | 0.00 | 0.00 | 0.00 | 0.00 | 0.00 | 0.00 | 0.00 | 0.00 | 0.00 | 0.00 | -0.01 | 0.00 | 0.00 |
| ocountry43 | 0.00 | 0.00 | 0.00 | 0.00 | 0.00 | -0.01 | 0.00 | -0.01 | -0.01 | 0.00 | 0.00 | 0.00 | 0.00 | -0.02 | 0.00 | 0.00 |
| ocountry44 | 0.00 | 0.00 | 0.00 | 0.00 | 0.00 | 0.00 | 0.00 | 0.00 | 0.00 | 0.00 | 0.00 | 0.00 | 0.00 | -0.01 | 0.00 | 0.00 |
| ocountry45 | 0.00 | 0.00 | 0.00 | 0.00 | 0.00 | 0.00 | 0.00 | 0.00 | 0.00 | 0.00 | 0.00 | 0.00 | 0.00 | -0.01 | 0.00 | 0.00 |
| ocountry46 | 0.00 | 0.00 | 0.00 | 0.00 | 0.00 | -0.01 | 0.00 | -0.01 | -0.01 | 0.00 | 0.00 | 0.00 | 0.00 | -0.02 | 0.00 | 0.00 |
| ocountry47 | -0.02 | -0.01 | -0.02 | -0.02 | -0.02 | -0.03 | -0.02 | -0.03 | -0.04 | -0.01 | -0.02 | -0.01 | -0.02 | -0.09 | -0.02 | -0.01 |
| ocountry48 | -0.01 | -0.01 | -0.01 | -0.01 | -0.01 | -0.02 | -0.01 | -0.02 | -0.03 | -0.01 | -0.01 | -0.01 | -0.01 | -0.07 | -0.02 | -0.01 |
|  | ocountry17 | ocountry18 | ocountry19 | ocountry20 | ocountry21 | ocountry22 | ocountry23 | ocountry24 | ocountry25 | ocountry26 | ocountry27 | ocountry28 | ocountry29 | ocountry30 | ocountry31 | ocountry32 |
| ocountry17 | 1.00 |  |  |  |  |  |  |  |  |  |  |  |  |  |  |  |
| ocountry18 | 0.00 | 1.00 |  |  |  |  |  |  |  |  |  |  |  |  |  |  |
| ocountry19 | 0.00 | 0.00 | 1.00 |  |  |  |  |  |  |  |  |  |  |  |  |  |
| ocountry20 | 0.00 | 0.00 | 0.00 | 1.00 |  |  |  |  |  |  |  |  |  |  |  |  |
| ocountry21 | -0.01 | -0.01 | -0.01 | -0.01 | 1.00 |  |  |  |  |  |  |  |  |  |  |  |
| ocountry22 | 0.00 | 0.00 | 0.00 | 0.00 | -0.01 | 1.00 |  |  |  |  |  |  |  |  |  |  |
| ocountry23 | 0.00 | 0.00 | 0.00 | 0.00 | 0.00 | 0.00 | 1.00 |  |  |  |  |  |  |  |  |  |
| ocountry24 | 0.00 | 0.00 | 0.00 | 0.00 | 0.00 | 0.00 | 0.00 | 1.00 |  |  |  |  |  |  |  |  |
| ocountry25 | 0.00 | 0.00 | 0.00 | 0.00 | -0.01 | 0.00 | 0.00 | 0.00 | 1.00 |  |  |  |  |  |  |  |
| ocountry26 | 0.00 | 0.00 | 0.00 | 0.00 | -0.01 | 0.00 | 0.00 | 0.00 | 0.00 | 1.00 |  |  |  |  |  |  |
| ocountry27 | 0.00 | 0.00 | 0.00 | 0.00 | 0.00 | 0.00 | 0.00 | 0.00 | 0.00 | 0.00 | 1.00 |  |  |  |  |  |
| ocountry28 | 0.00 | 0.00 | 0.00 | 0.00 | -0.01 | 0.00 | 0.00 | 0.00 | 0.00 | 0.00 | 0.00 | 1.00 |  |  |  |  |
| ocountry29 | 0.00 | 0.00 | 0.00 | 0.00 | 0.00 | 0.00 | 0.00 | 0.00 | 0.00 | 0.00 | 0.00 | 0.00 | 1.00 |  |  |  |
| ocountry30 | 0.00 | 0.00 | -0.01 | 0.00 | -0.01 | 0.00 | 0.00 | 0.00 | 0.00 | 0.00 | 0.00 | 0.00 | 0.00 | 1.00 |  |  |
| ocountry31 | 0.00 | 0.00 | 0.00 | 0.00 | 0.00 | 0.00 | 0.00 | 0.00 | 0.00 | 0.00 | 0.00 | 0.00 | 0.00 | 0.00 | 1.00 |  |
| ocountry32 | 0.00 | 0.00 | -0.01 | 0.00 | -0.01 | 0.00 | 0.00 | 0.00 | 0.00 | 0.00 | 0.00 | 0.00 | 0.00 | -0.01 | 0.00 | 1.00 |
|  | ocountry17 | ocountry18 | ocountry19 | ocountry20 | ocountry21 | ocountry22 | ocountry23 | ocountry24 | ocountry25 | ocountry26 | ocountry27 | ocountry28 | ocountry29 | ocountry30 | ocountry31 | ocountry32 |
| ocountry33 | -0.01 | -0.01 | -0.01 | -0.01 | -0.02 | -0.01 | 0.00 | 0.00 | -0.01 | 0.00 | 0.00 | -0.01 | 0.00 | -0.01 | 0.00 | -0.01 |
| ocountry34 | 0.00 | 0.00 | 0.00 | 0.00 | 0.00 | 0.00 | 0.00 | 0.00 | 0.00 | 0.00 | 0.00 | 0.00 | 0.00 | 0.00 | 0.00 | 0.00 |
| ocountry35 | -0.06 | -0.05 | -0.07 | -0.05 | -0.17 | -0.06 | -0.03 | -0.03 | -0.06 | -0.04 | -0.03 | -0.05 | -0.03 | -0.08 | -0.03 | -0.08 |
| ocountry36 | 0.00 | 0.00 | 0.00 | 0.00 | -0.01 | 0.00 | 0.00 | 0.00 | 0.00 | 0.00 | 0.00 | 0.00 | 0.00 | 0.00 | 0.00 | 0.00 |
| ocountry37 | 0.00 | 0.00 | 0.00 | 0.00 | -0.01 | 0.00 | 0.00 | 0.00 | 0.00 | 0.00 | 0.00 | 0.00 | 0.00 | 0.00 | 0.00 | 0.00 |
| ocountry38 | -0.01 | -0.01 | -0.01 | -0.01 | -0.04 | -0.01 | -0.01 | -0.01 | -0.01 | -0.01 | -0.01 | -0.01 | -0.01 | -0.02 | -0.01 | -0.02 |
| ocountry39 | 0.00 | 0.00 | 0.00 | 0.00 | 0.00 | 0.00 | 0.00 | 0.00 | 0.00 | 0.00 | 0.00 | 0.00 | 0.00 | 0.00 | 0.00 | 0.00 |
| ocountry40 | 0.00 | 0.00 | 0.00 | 0.00 | -0.01 | 0.00 | 0.00 | 0.00 | 0.00 | 0.00 | 0.00 | 0.00 | 0.00 | 0.00 | 0.00 | 0.00 |
| ocountry41 | 0.00 | 0.00 | -0.01 | 0.00 | -0.01 | 0.00 | 0.00 | 0.00 | 0.00 | 0.00 | 0.00 | 0.00 | 0.00 | -0.01 | 0.00 | -0.01 |
| ocountry42 | 0.00 | 0.00 | 0.00 | 0.00 | 0.00 | 0.00 | 0.00 | 0.00 | 0.00 | 0.00 | 0.00 | 0.00 | 0.00 | 0.00 | 0.00 | 0.00 |
| ocountry43 | 0.00 | 0.00 | -0.01 | 0.00 | -0.01 | 0.00 | 0.00 | 0.00 | 0.00 | 0.00 | 0.00 | 0.00 | 0.00 | -0.01 | 0.00 | -0.01 |
| ocountry44 | 0.00 | 0.00 | 0.00 | 0.00 | 0.00 | 0.00 | 0.00 | 0.00 | 0.00 | 0.00 | 0.00 | 0.00 | 0.00 | 0.00 | 0.00 | 0.00 |
| ocountry45 | 0.00 | 0.00 | 0.00 | 0.00 | 0.00 | 0.00 | 0.00 | 0.00 | 0.00 | 0.00 | 0.00 | 0.00 | 0.00 | 0.00 | 0.00 | 0.00 |
| ocountry46 | 0.00 | 0.00 | -0.01 | 0.00 | -0.01 | 0.00 | 0.00 | 0.00 | 0.00 | 0.00 | 0.00 | 0.00 | 0.00 | -0.01 | 0.00 | -0.01 |
| ocountry47 | -0.02 | -0.02 | -0.03 | -0.02 | -0.07 | -0.02 | -0.01 | -0.01 | -0.02 | -0.02 | -0.01 | -0.02 | -0.01 | -0.03 | -0.01 | -0.03 |
| ocountry48 | -0.02 | -0.01 | -0.02 | -0.01 | -0.05 | -0.02 | -0.01 | -0.01 | -0.02 | -0.01 | -0.01 | -0.01 | -0.01 | -0.02 | -0.01 | -0.02 |
|  | ocountry33 | ocountry34 | ocountry35 | ocountry36 | ocountry37 | ocountry38 | ocountry39 | ocountry40 | ocountry41 | ocountry42 | ocountry43 | ocountry44 | ocountry45 | ocountry46 | ocountry47 | ocountry48 |
| ocountry33 | 1.00 |  |  |  |  |  |  |  |  |  |  |  |  |  |  |  |
| ocountry34 | 0.00 | 1.00 |  |  |  |  |  |  |  |  |  |  |  |  |  |  |
| ocountry35 | -0.12 | -0.03 | 1.00 |  |  |  |  |  |  |  |  |  |  |  |  |  |
| ocountry36 | -0.01 | 0.00 | -0.06 | 1.00 |  |  |  |  |  |  |  |  |  |  |  |  |
| ocountry37 | -0.01 | 0.00 | -0.05 | 0.00 | 1.00 |  |  |  |  |  |  |  |  |  |  |  |
| ocountry38 | -0.03 | -0.01 | -0.23 | -0.01 | -0.01 | 1.00 |  |  |  |  |  |  |  |  |  |  |
| ocountry39 | 0.00 | 0.00 | -0.03 | 0.00 | 0.00 | -0.01 | 1.00 |  |  |  |  |  |  |  |  |  |
| ocountry40 | -0.01 | 0.00 | -0.05 | 0.00 | 0.00 | -0.01 | 0.00 | 1.00 |  |  |  |  |  |  |  |  |
| ocountry41 | -0.01 | 0.00 | -0.08 | 0.00 | 0.00 | -0.02 | 0.00 | 0.00 | 1.00 |  |  |  |  |  |  |  |
| ocountry42 | 0.00 | 0.00 | -0.03 | 0.00 | 0.00 | -0.01 | 0.00 | 0.00 | 0.00 | 1.00 |  |  |  |  |  |  |
| ocountry43 | -0.01 | 0.00 | -0.09 | 0.00 | 0.00 | -0.02 | 0.00 | 0.00 | -0.01 | 0.00 | 1.00 |  |  |  |  |  |
| ocountry44 | 0.00 | 0.00 | -0.03 | 0.00 | 0.00 | -0.01 | 0.00 | 0.00 | 0.00 | 0.00 | 0.00 | 1.00 |  |  |  |  |
| ocountry45 | 0.00 | 0.00 | -0.03 | 0.00 | 0.00 | -0.01 | 0.00 | 0.00 | 0.00 | 0.00 | 0.00 | 0.00 | 1.00 |  |  |  |
| ocountry46 | -0.01 | 0.00 | -0.09 | 0.00 | 0.00 | -0.02 | 0.00 | 0.00 | -0.01 | 0.00 | -0.01 | 0.00 | 0.00 | 1.00 |  |  |
| ocountry47 | -0.05 | -0.01 | -0.45 | -0.02 | -0.02 | -0.09 | -0.01 | -0.02 | -0.03 | -0.01 | -0.04 | -0.01 | -0.01 | -0.04 | 1.00 |  |
| ocountry48 | -0.04 | -0.01 | -0.33 | -0.02 | -0.01 | -0.07 | -0.01 | -0.01 | -0.02 | -0.01 | -0.03 | -0.01 | -0.01 | -0.03 | -0.13 | 1.00 |

Note: 1, The output based on 1314 obviations contains: the Pearson correlation coefficient "r" is shown in the first row; 2, We follow the critical value of general guidelines that provided by [SI.1] Cohen (1988) [SI.1] on | r | > .5 as large/strong correlation and no strong correlation was found from the coefficient.

**S5A Table Regression results of key control variables: Standard errors, p value and 95% Confident Interval.**

|  |  |  |  |  |  |  |  |  |  |  |  |  |  |  |
| --- | --- | --- | --- | --- | --- | --- | --- | --- | --- | --- | --- | --- | --- | --- |
| Model ID | 1 |  | 2 |  | 3 |  | 4 |  | 5 |  | 6 |  | 7 |  |
|  | 11Mar |  | 13Mar |  | 16Mar |  | 17Mar |  | 19Mar |  | pfl |  | 21Mar |  |
| Variable name | Std. Err. | P>\|z\| | Std. Err. | P>\|z\| | Std. Err. | P>\|z\| | Std. Err. | P>\|z\| | Std. Err. | P>\|z\| | Std. Err. | P>\|z\| | Std. Err. | P>\|z\| |
| 1.v2 | (0.09) | (0.008) | (0.09) | (0.010) | (0.09) | (0.010) | (0.09) | (0.012) | (0.09) | (0.013) | (0.10) | (0.041) | (0.10) | (0.036) |
| *95% CI* | [0.07 | 0.43] | [0.06 | 0.43] | [0.06 | 0.43] | [0.05 | 0.42] | [0.05 | 0.42] | [0.01 | 0.38] | [0.01 | 0.39] |
| 1.v9 | (0.06) | (0.001) | (0.06) | (0.001) | (0.06) | (0.001) | (0.06) | (0.001) | (0.06) | (0.001) | (0.06) | (0.003) | (0.06) | (0.003) |
|  | [-0.31 | -0.08] | [-0.31 | -0.08] | [-0.31 | -0.08] | [-0.30 | -0.07] | [-0.30 | -0.07] | [-0.29 | -0.06] | [-0.29 | -0.06] |
| 1.v12 | (0.08) | (0.000) | (0.09) | (0.000) | (0.09) | (0.000) | (0.09) | (0.000) | (0.09) | (0.000) | (0.08) | (0.000) | (0.08) | (0.000) |
|  | [-0.73 | -0.40] | [-0.73 | -0.40] | [-0.73 | -0.40] | [-0.75 | -0.41] | [-0.75 | -0.41] | [-0.74 | -0.41] | [-0.74 | -0.41] |
| v14 | (0.06) | (0.626) | (0.06) | (0.655) | (0.06) | (0.610) | (0.06) | (0.461) | (0.06) | (0.460) | (0.06) | (0.511) | (0.06) | (0.501) |
|  | [-0.14 | 0.08] | [-0.14 | 0.09] | [-0.14 | 0.08] | [-0.16 | 0.07] | [-0.16 | 0.07] | [-0.16 | 0.08] | [-0.16 | 0.08] |
| 2.v4 | (0.49) | (0.000) | (0.49) | (0.000) | (0.49) | (0.000) | (0.51) | (0.000) | (0.51) | (0.000) | (0.52) | (0.000) | (1.19) | (0.009) |
|  | [-3.10 | -1.19] | [-3.07 | -1.16] | [-3.09 | -1.18] | [-3.29 | -1.28] | [-3.29 | -1.28] | [-3.49 | -1.44] | [-5.47 | -0.79] |
| v5_1 | (0.16) | (0.000) | (0.16) | (0.000) | (0.16) | (0.000) | (0.16) | (0.000) | (0.16) | (0.000) | (0.16) | (0.000) | (0.39) | (0.031) |
|  | [0.27 | 0.88] | [0.26 | 0.87] | [0.26 | 0.87] | [0.27 | 0.89] | [0.27 | 0.89] | [0.31 | 0.93] | [0.08 | 1.61] |
| 2.v6 | (0.58) | (0.000) | (0.58) | (0.000) | (0.59) | (0.000) | (0.60) | (0.001) | (0.60) | (0.001) | (0.61) | (0.000) | (1.35) | (0.023) |
|  | [0.92 | 3.19] | [0.90 | 3.18] | [0.97 | 3.28] | [0.85 | 3.21] | [0.85 | 3.21] | [1.11 | 3.50] | [0.42 | 5.70] |
| 3.v6 | (0.30) | (0.004) | (0.30) | (0.004) | (0.32) | (0.003) | (0.33) | (0.008) | (0.33) | (0.008) | (0.33) | (0.004) | (0.34) | (0.004) |
|  | [0.29 | 1.46] | [0.28 | 1.45] | [0.34 | 1.57] | [0.23 | 1.53] | [0.23 | 1.52] | [0.30 | 1.61] | [0.32 | 1.66] |
| v7 | (0.00) | (0.000) | (0.00) | (0.000) | (0.00) | (0.000) | (0.00) | (0.000) | (0.00) | (0.000) | (0.00) | (0.002) | (0.00) | (0.002) |
|  | [-0.01 | -0.01] | [-0.01 | -0.01] | [-0.01 | -0.01] | [-0.01 | -0.01] | [-0.01 | -0.01] | [-0.01 | -0.00] | [-0.01 | -0.00] |
|  |  |  |  |  |  |  |  |  |  |  |  |  |  |  |
| Model ID | 8 |  | 9 |  | 10 |  | 11 |  | 12 |  | 13 |  | 14 |  |
| Variable name | pfr |  | 25Mar |  | 26Mar |  | 27Mar |  | 28Mar |  | 2Apr |  | 7Apr |  |
| 1.v2 | (0.10) | (0.036) | (0.10) | (0.039) | (0.09) | (0.029) | (0.09) | (0.021) | (0.09) | (0.023) | (0.09) | (0.013) | (0.09) | (0.015) |
|  | [0.01 | 0.39] | [0.01 | 0.39] | [0.02 | 0.39] | [0.03 | 0.40] | [0.03 | 0.40] | [0.05 | 0.42] | [0.05 | 0.42] |
| 1.v9 | (0.06) | (0.003) | (0.06) | (0.003) | (0.06) | (0.003) | (0.06) | (0.003) | (0.06) | (0.003) | (0.06) | (0.008) | (0.05) | (0.001) |
|  | [-0.29 | -0.06] | [-0.29 | -0.06] | [-0.29 | -0.06] | [-0.29 | -0.06] | [-0.29 | -0.06] | [-0.26 | -0.04] | [-0.29 | -0.07] |
| 1.v12 | (0.08) | (0.000) | (0.08) | (0.000) | (0.08) | (0.000) | (0.08) | (0.000) | (0.09) | (0.000) | (0.08) | (0.000) | (0.08) | (0.000) |
|  | [-0.74 | -0.41] | [-0.74 | -0.41] | [-0.76 | -0.43] | [-0.74 | -0.41] | [-0.76 | -0.42] | [-0.78 | -0.45] | [-0.78 | -0.45] |
| v14 | (0.06) | (0.521) | (0.06) | (0.579) | (0.06) | (0.760) | (0.06) | (0.879) | (0.06) | (0.923) | (0.06) | (0.745) | (0.06) | (0.681) |
|  | [-0.16 | 0.08] | [-0.16 | 0.09] | [-0.14 | 0.10] | [-0.13 | 0.11] | [-0.13 | 0.12] | [-0.14 | 0.10] | [-0.14 | 0.09] |
| 2.v4 | (1.21) | (0.010) | (1.21) | (0.011) | (1.16) | (0.021) | (1.15) | (0.021) | (1.31) | (0.021) | (1.34) | (0.028) | (1.33) | (0.028) |
|  | [-5.50 | -0.75] | [-5.45 | -0.71] | [-4.96 | -0.41] | [-4.91 | -0.40] | [-5.60 | -0.46] | [-5.55 | -0.32] | [-5.52 | -0.32] |
| v5_1 | (0.40) | (0.032) | (0.39) | (0.034) | (0.38) | (0.142) | (0.38) | (0.135) | (0.42) | (0.120) | (0.43) | (0.109) | (0.43) | (0.111) |
|  | [0.07 | 1.62] | [0.06 | 1.61] | [-0.19 | 1.31] | [-0.18 | 1.32] | [-0.17 | 1.49] | [-0.15 | 1.54] | [-0.16 | 1.53] |
| 2.v6 | (1.36) | (0.025) | (1.35) | (0.028) | (1.29) | (0.054) | (1.31) | (0.042) | (1.41) | (0.044) | (1.44) | (0.022) | (1.43) | (0.019) |
|  | [0.38 | 5.70] | [0.32 | 5.62] | [-0.04 | 5.03] | [0.10 | 5.22] | [0.08 | 5.62] | [0.46 | 6.10] | [0.55 | 6.16] |
| 3.v6 | (0.35) | (0.005) | (0.35) | (0.008) | (0.37) | (0.105) | (0.37) | (0.127) | (0.37) | (0.194) | (0.37) | (0.103) | (0.37) | (0.109) |
|  | [0.29 | 1.66] | [0.24 | 1.62] | [-0.13 | 1.33] | [-0.16 | 1.28] | [-0.25 | 1.22] | [-0.12 | 1.34] | [-0.13 | 1.33] |
| v7 | (0.00) | (0.003) | (0.00) | (0.003) | (0.00) | (0.000) | (0.00) | (0.000) | (0.00) | (0.000) | (0.00) | (0.049) | (0.00) | (0.026) |
|  | [-0.01 | -0.00] | [-0.01 | -0.00] | [-0.02 | -0.01] | [-0.02 | -0.01] | [-0.02 | -0.01] | [-0.01 | -0.00] | [-0.01 | -0.00] |
| N=observation | 1176 |  | 1176 |  | 1176 |  | 1176 |  | 1176 |  | 1176 |  | 1176 |  |

Note: the second line of every variable row reports the 95% Confidence interval.

**S5B Table Regression results of policy variables: Standard errors, p value and 95% Confident Interval.**

| Model ID | 1 |  | 2 |  | 3 |  | 4 |  | 5 |  | 6 |  | 7 |  |
| --- | --- | --- | --- | --- | --- | --- | --- | --- | --- | --- | --- | --- | --- | --- |
|  | 11Mar |  | 13Mar |  | 16Mar |  | 17Mar |  | 19Mar |  | pfl |  | 21Mar |  |
| Variable name | Std. Err. | P>\|z\| | Std. Err. | P>\|z\| | Std. Err. | P>\|z\| | Std. Err. | P>\|z\| | Std. Err. | P>\|z\| | Std. Err. | P>\|z\| | Std. Err. | P>\|z\| |
| 1.p311bjr | (0.33) | (0.360) | (0.33) | (0.345) | (0.35) | (0.207) | (0.35) | (0.243) | (0.35) | (0.245) | (0.35) | (0.218) | (0.36) | (0.208) |
|  | [-0.35 | 0.96] | [-0.34 | 0.97] | [-0.25 | 1.14] | [-0.28 | 1.11] | [-0.28 | 1.11] | [-0.26 | 1.13] | [-0.25 | 1.15] |
| 1.p313shr |  |  | (0.28) | (0.389) | (0.28) | (0.364) | (0.29) | (0.382) | (0.29) | (0.384) | (0.29) | (0.562) | (0.29) | (0.570) |
|  |  |  | [-0.31 | 0.79] | [-0.30 | 0.81] | [-0.32 | 0.82] | [-0.32 | 0.82] | [-0.40 | 0.74] | [-0.40 | 0.73] |
| 1.p316bjr |  |  |  |  | (0.27) | (0.273) | (0.27) | (0.344) | (0.27) | (0.343) | (0.28) | (0.501) | (0.28) | (0.462) |
|  |  |  |  |  | [-0.24 | 0.84] | [-0.28 | 0.80] | [-0.28 | 0.80] | [-0.35 | 0.72] | [-0.34 | 0.75] |
| 1.p317shr |  |  |  |  |  |  | (0.11) | (0.479) | (0.11) | (0.470) | (0.12) | (0.641) | (0.12) | (0.622) |
|  |  |  |  |  |  |  | [-0.14 | 0.29] | [-0.14 | 0.29] | [-0.30 | 0.18] | [-0.30 | 0.18] |
| 1.p317ynr |  |  |  |  |  |  | (0.50) | (0.001) | (0.50) | (0.001) | (0.49) | (0.001) | (0.49) | (0.001) |
|  |  |  |  |  |  |  | [0.72 | 2.67] | [0.73 | 2.68] | [0.68 | 2.60] | [0.67 | 2.59] |
| 1.p317xar |  |  |  |  |  |  | (0.31) | (0.018) | (0.31) | (0.017) | (0.31) | (0.052) | (0.31) | (0.048) |
|  |  |  |  |  |  |  | [0.13 | 1.34] | [0.13 | 1.34] | [-0.01 | 1.22] | [0.00 | 1.21] |
| 1.p319gdr |  |  |  |  |  |  |  |  | (0.16) | (0.796) | (0.17) | (0.511) | (0.17) | (0.523) |
|  |  |  |  |  |  |  |  |  | [-0.27 | 0.35] | [-0.44 | 0.22] | [-0.44 | 0.23] |
| 2.pfl |  |  |  |  |  |  |  |  |  |  | (0.12) | (0.741) | (0.12) | (0.773) |
|  |  |  |  |  |  |  |  |  |  |  | [-0.20 | 0.28] | [-0.20 | 0.27] |
| 3.pfl |  |  |  |  |  |  |  |  |  |  | (0.15) | (0.123) | (0.15) | (0.116) |
|  |  |  |  |  |  |  |  |  |  |  | [-0.54 | 0.06] | [-0.55 | 0.06] |
| 1.p321tj |  |  |  |  |  |  |  |  |  |  |  |  | (0.72) | (0.525) |
|  |  |  |  |  |  |  |  |  |  |  |  |  | [-0.96 | 1.87] |
| 1.pfr |  |  |  |  |  |  |  |  |  |  |  |  |  |  |
|  |  |  |  |  |  |  |  |  |  |  |  |  |  |  |
| 2.pfr |  |  |  |  |  |  |  |  |  |  |  |  |  |  |
|  |  |  |  |  |  |  |  |  |  |  |  |  |  |  |
| 1.p325bj |  |  |  |  |  |  |  |  |  |  |  |  |  |  |
|  |  |  |  |  |  |  |  |  |  |  |  |  |  |  |
| 1.p326sh |  |  |  |  |  |  |  |  |  |  |  |  |  |  |
|  |  |  |  |  |  |  |  |  |  |  |  |  |  |  |
| 1.p327yn |  |  |  |  |  |  |  |  |  |  |  |  |  |  |
|  |  |  |  |  |  |  |  |  |  |  |  |  |  |  |
| 1.p327gd |  |  |  |  |  |  |  |  |  |  |  |  |  |  |
|  |  |  |  |  |  |  |  |  |  |  |  |  |  |  |
| 1.p328xa |  |  |  |  |  |  |  |  |  |  |  |  |  |  |
|  |  |  |  |  |  |  |  |  |  |  |  |  |  |  |
| 1.p402hlj |  |  |  |  |  |  |  |  |  |  |  |  |  |  |
|  |  |  |  |  |  |  |  |  |  |  |  |  |  |  |
| 1.p407hlj |  |  |  |  |  |  |  |  |  |  |  |  |  |  |
| Model ID | 8 |  | 9 |  | 10 |  | 11 |  | 12 |  | 13 |  | 14 |  |
|  | pfr |  | 25Mar |  | 26Mar |  | 27Mar |  | 28Mar |  | 2Apr |  | 7Apr |  |
| Variable name | Std. Err. | P>\|z\| | Std. Err. | P>\|z\| | Std. Err. | P>\|z\| | Std. Err. | P>\|z\| | Std. Err. | P>\|z\| | Std. Err. | P>\|z\| | Std. Err. | P>\|z\| |
| 1.p311bjr | (0.36) | (0.220) | (0.36) | (0.242) | (0.36) | (0.270) | (0.36) | (0.313) | (0.36) | (0.342) | (0.36) | (0.196) | (0.36) | (0.200) |
|  | [-0.26 | 1.14] | [-0.28 | 1.12] | [-0.31 | 1.10] | [-0.34 | 1.07] | [-0.36 | 1.04] | [-0.24 | 1.16] | [-0.24 | 1.15] |
| 1.p313shr | (0.29) | (0.560) | (0.29) | (0.561) | (0.31) | (0.057) | (0.31) | (0.058) | (0.31) | (0.059) | (0.31) | (0.070) | (0.31) | (0.069) |
|  | [-0.40 | 0.74] | [-0.40 | 0.74] | [-0.02 | 1.20] | [-0.02 | 1.20] | [-0.02 | 1.20] | [-0.05 | 1.17] | [-0.04 | 1.17] |
| 1.p316bjr | (0.28) | (0.459) | (0.29) | (0.591) | (0.29) | (0.424) | (0.29) | (0.352) | (0.29) | (0.385) | (0.28) | (0.494) | (0.28) | (0.478) |
|  | [-0.34 | 0.76] | [-0.41 | 0.72] | [-0.34 | 0.80] | [-0.30 | 0.85] | [-0.32 | 0.82] | [-0.36 | 0.75] | [-0.36 | 0.76] |
| 1.p317shr | (0.13) | (0.643) | (0.13) | (0.635) | (0.17) | (0.031) | (0.17) | (0.017) | (0.17) | (0.021) | (0.17) | (0.036) | (0.17) | (0.035) |
|  | [-0.30 | 0.19] | [-0.31 | 0.19] | [0.03 | 0.69] | [0.07 | 0.75] | [0.06 | 0.73] | [0.02 | 0.70] | [0.03 | 0.70] |
| 1.p317ynr | (0.49) | (0.001) | (0.49) | (0.001) | (0.47) | (0.001) | (0.49) | (0.001) | (0.49) | (0.001) | (0.49) | (0.003) | (0.48) | (0.003) |
|  | [0.67 | 2.58] | [0.67 | 2.59] | [0.68 | 2.53] | [0.62 | 2.56] | [0.63 | 2.57] | [0.50 | 2.41] | [0.50 | 2.39] |
| 1.p317xar | (0.32) | (0.059) | (0.32) | (0.059) | (0.32) | (0.171) | (0.33) | (0.289) | (0.68) | (0.875) | (0.65) | (0.753) | (0.65) | (0.763) |
|  | [-0.02 | 1.22] | [-0.02 | 1.22] | [-0.19 | 1.06] | [-0.29 | 0.99] | [-1.44 | 1.23] | [-1.07 | 1.48] | [-1.08 | 1.47] |
| 1.p319gdr | (0.17) | (0.519) | (0.17) | (0.534) | (0.17) | (0.683) | (0.21) | (0.509) | (0.21) | (0.544) | (0.21) | (0.806) | (0.21) | (0.834) |
|  | [-0.44 | 0.22] | [-0.44 | 0.23] | [-0.40 | 0.26] | [-0.28 | 0.56] | [-0.29 | 0.55] | [-0.47 | 0.36] | [-0.46 | 0.37] |
| 2.pfl | (0.12) | (0.770) | (0.12) | (0.732) | (0.12) | (0.740) | (0.12) | (0.996) | (0.12) | (0.942) | (0.12) | (0.539) | (0.12) | (0.639) |
|  | [-0.20 | 0.27] | [-0.20 | 0.28] | [-0.19 | 0.27] | [-0.24 | 0.24] | [-0.23 | 0.25] | [-0.31 | 0.16] | [-0.29 | 0.18] |
| 3.pfl | (0.16) | (0.115) | (0.16) | (0.127) | (0.16) | (0.021) | (0.16) | (0.007) | (0.16) | (0.011) | (0.16) | (0.005) | (0.16) | (0.007) |
|  | [-0.55 | 0.06] | [-0.55 | 0.07] | [-0.66 | -0.06] | [-0.75 | -0.12] | [-0.73 | -0.09] | [-0.75 | -0.13] | [-0.73 | -0.11] |
| 1.p321tj | (0.75) | (0.529) | (0.75) | (0.546) | (0.70) | (0.651) | (0.71) | (0.576) | (0.77) | (0.509) | (0.78) | (0.455) | (0.77) | (0.449) |
|  | [-1.00 | 1.94] | [-1.01 | 1.92] | [-1.06 | 1.70] | [-0.99 | 1.78] | [-1.00 | 2.02] | [-0.94 | 2.10] | [-0.93 | 2.10] |
| 1.pfr | (0.38) | (0.785) | (0.40) | (0.921) | (0.41) | (0.762) | (0.41) | (0.690) | (0.41) | (0.691) |  |  |  |  |
|  | [-0.85 | 0.64] | [-0.75 | 0.83] | [-0.68 | 0.92] | [-0.64 | 0.97] | [-0.64 | 0.96] |  |  |  |  |
| 2.pfr | (0.13) | (0.914) | (0.13) | (0.916) | (0.14) | (0.196) | (0.14) | (0.088) | (0.15) | (0.061) |  |  |  |  |
|  | [-0.27 | 0.24] | [-0.27 | 0.24] | [-0.09 | 0.45] | [-0.04 | 0.53] | [-0.01 | 0.59] |  |  |  |  |
| 1.p325bj |  |  | (0.95) | (0.464) | (0.95) | (0.465) | (0.95) | (0.421) | (0.94) | (0.405) | (0.87) | (0.403) | (0.87) | (0.399) |
|  |  |  | [-2.55 | 1.16] | [-2.55 | 1.17] | [-2.63 | 1.10] | [-2.64 | 1.07] | [-2.44 | 0.98] | [-2.44 | 0.97] |
| 1.p326sh |  |  |  |  | (0.19) | (0.000) | (0.21) | (0.000) | (0.21) | (0.000) | (0.20) | (0.004) | (0.20) | (0.003) |
|  |  |  |  |  | [0.41 | 1.17] | [0.51 | 1.34] | [0.50 | 1.33] | [0.18 | 0.95] | [0.19 | 0.95] |
| 1.p327yn |  |  |  |  |  |  | (1.22) | (0.957) | (1.22) | (0.956) | (1.23) | (0.771) | (1.23) | (0.750) |
|  |  |  |  |  |  |  | [-2.33 | 2.47] | [-2.33 | 2.47] | [-2.76 | 2.05] | [-2.79 | 2.01] |
| 1.p327gd |  |  |  |  |  |  | (0.22) | (0.105) | (0.22) | (0.125) | (0.21) | (0.847) | (0.21) | (0.803) |
|  |  |  |  |  |  |  | [-0.07 | 0.78] | [-0.09 | 0.76] | [-0.38 | 0.46] | [-0.36 | 0.47] |
| 1.p328xa |  |  |  |  |  |  |  |  | (0.63) | (0.446) | (0.59) | (0.676) | (0.59) | (0.672) |
|  |  |  |  |  |  |  |  |  | [-1.72 | 0.76] | [-1.41 | 0.91] | [-1.40 | 0.90] |
| 1.p402hlj |  |  |  |  |  |  |  |  |  |  | (0.07) | (0.000) | (0.07) | (0.000) |
|  |  |  |  |  |  |  |  |  |  |  | [-0.78 | -0.50] | [-0.73 | -0.45] |
| 1.p407hlj |  |  |  |  |  |  |  |  |  |  |  |  | (0.15) | (0.000) |
|  |  |  |  |  |  |  |  |  |  |  |  |  | [-0.99 | -0.39] |
| N=observation | 1176 |  | 1176 |  | 1176 |  | 1176 |  | 1176 |  | 1176 |  | 1176 |  |

Note: the second line of every variable row reports the 95% Confidence interval

**S5C Table Regression results of country variables: Standard errors, p value, marginal effect and 95% Confident Interval.**

| Model ID | 1 |  | 2 |  | 3 |  | 4 |  | 5 |  | 6 |  | 7 |  |
| --- | --- | --- | --- | --- | --- | --- | --- | --- | --- | --- | --- | --- | --- | --- |
|  | 11Mar |  | 13Mar |  | 16Mar |  | 17Mar |  | 19Mar |  | pfl |  | 21Mar |  |
| Variable name | Std. Err. | P>\|z\| | Std. Err. | P>\|z\| | Std. Err. | P>\|z\| | Std. Err. | P>\|z\| | Std. Err. | P>\|z\| | Std. Err. | P>\|z\| | Std. Err. | P>\|z\| |
| ocountry1 | (0.32) | (0.375) | (0.32) | (0.351) | (0.32) | (0.355) | (0.32) | (0.342) | (0.32) | (0.332) | (0.35) | (0.472) | (0.35) | (0.467) |
| ocountry2 | (0.38) | (0.000) | (0.38) | (0.000) | (0.38) | (0.000) | (0.38) | (0.000) | (0.38) | (0.000) | (0.38) | (0.000) | (0.38) | (0.000) |
| ocountry4 | (0.51) | (0.883) | (0.51) | (0.859) | (0.51) | (0.884) | (0.51) | (0.559) | (0.51) | (0.546) | (0.51) | (0.486) | (0.51) | (0.480) |
| ocountry5 | (0.38) | (0.049) | (0.38) | (0.051) | (0.38) | (0.035) | (0.38) | (0.019) | (0.38) | (0.019) | (0.38) | (0.022) | (0.38) | (0.020) |
| ocountry6 | (0.30) | (0.597) | (0.30) | (0.578) | (0.30) | (0.553) | (0.30) | (0.445) | (0.30) | (0.443) | (0.29) | (0.361) | (0.29) | (0.367) |
| ocountry7 | (0.37) | (0.886) | (0.37) | (0.902) | (0.37) | (0.919) | (0.37) | (0.975) | (0.37) | (0.989) | (0.37) | (0.730) | (0.37) | (0.728) |
| ocountry8 | (0.36) | (0.343) | (0.36) | (0.342) | (0.36) | (0.353) | (0.35) | (0.356) | (0.35) | (0.370) | (0.36) | (0.426) | (0.36) | (0.438) |
| ocountry9 | (0.22) | (0.214) | (0.22) | (0.221) | (0.22) | (0.190) | (0.22) | (0.172) | (0.23) | (0.166) | (0.23) | (0.232) | (0.23) | (0.221) |
| ocountry10 | (0.59) | (0.871) | (0.59) | (0.880) | (0.59) | (0.888) | (0.59) | (0.896) | (0.59) | (0.911) | (0.59) | (0.751) | (0.59) | (0.760) |
| ocountry11 | (0.41) | (0.094) | (0.41) | (0.103) | (0.41) | (0.101) | (0.40) | (0.100) | (0.41) | (0.105) | (0.42) | (0.088) | (0.43) | (0.092) |
| ocountry13 | (0.56) | (0.302) | (0.57) | (0.305) | (0.56) | (0.306) | (0.56) | (0.296) | (0.56) | (0.304) | (0.57) | (0.202) | (0.57) | (0.211) |
| ocountry14 | (0.14) | (0.361) | (0.14) | (0.514) | (0.14) | (0.517) | (0.15) | (0.301) | (0.15) | (0.296) | (0.15) | (0.261) | (0.15) | (0.275) |
| ocountry15 | (0.45) | (0.207) | (0.46) | (0.150) | (0.46) | (0.153) | (0.46) | (0.196) | (0.46) | (0.196) | (0.46) | (0.130) | (0.46) | (0.128) |
| ocountry17 | (0.82) | (0.661) | (0.82) | (0.657) | (0.83) | (0.774) | (0.83) | (0.775) | (0.83) | (0.775) | (0.83) | (0.712) | (0.83) | (0.734) |
| ocountry18 | (0.37) | (0.417) | (0.37) | (0.405) | (0.37) | (0.387) | (0.37) | (0.251) | (0.37) | (0.251) | (0.38) | (0.428) | (0.38) | (0.430) |
| ocountry19 | (0.34) | (0.118) | (0.34) | (0.131) | (0.34) | (0.135) | (0.35) | (0.209) | (0.35) | (0.207) | (0.35) | (0.161) | (0.35) | (0.155) |
| ocountry20 | (0.50) | (0.362) | (0.50) | (0.367) | (0.50) | (0.374) | (0.50) | (0.398) | (0.50) | (0.408) | (0.51) | (0.371) | (0.51) | (0.376) |
| ocountry21 | (0.23) | (0.166) | (0.23) | (0.171) | (0.23) | (0.193) | (0.23) | (0.234) | (0.23) | (0.237) | (0.26) | (0.148) | (0.26) | (0.142) |
| ocountry22 | (0.44) | (0.145) | (0.44) | (0.145) | (0.44) | (0.153) | (0.45) | (0.210) | (0.45) | (0.209) | (0.46) | (0.305) | (0.46) | (0.307) |
| ocountry24 | (0.45) | (0.526) | (0.45) | (0.508) | (0.45) | (0.508) | (0.44) | (0.497) | (0.44) | (0.485) | (0.46) | (0.611) | (0.46) | (0.607) |
| ocountry25 | (0.45) | (0.541) | (0.45) | (0.532) | (0.45) | (0.549) | (0.45) | (0.546) | (0.45) | (0.563) | (0.45) | (0.479) | (0.45) | (0.498) |
| ocountry26 | (1.01) | (0.107) | (1.01) | (0.111) | (1.01) | (0.111) | (1.01) | (0.123) | (1.01) | (0.123) | (1.02) | (0.106) | (1.02) | (0.105) |
| ocountry27 | (0.44) | (0.036) | (0.44) | (0.037) | (0.44) | (0.037) | (0.57) | (0.000) | (0.57) | (0.000) | (0.56) | (0.000) | (0.56) | (0.000) |
| ocountry28 | (0.50) | (0.601) | (0.50) | (0.623) | (0.50) | (0.627) | (0.50) | (0.706) | (0.50) | (0.704) | (0.50) | (0.675) | (0.50) | (0.665) |
| ocountry29 | (0.35) | (0.002) | (0.35) | (0.002) | (0.35) | (0.002) | (0.34) | (0.002) | (0.34) | (0.002) | (0.35) | (0.006) | (0.35) | (0.006) |
| ocountry30 | (0.19) | (0.007) | (0.19) | (0.006) | (0.19) | (0.006) | (0.19) | (0.005) | (0.20) | (0.005) | (0.20) | (0.016) | (0.20) | (0.016) |
| ocountry32 | (0.29) | (0.140) | (0.29) | (0.144) | (0.29) | (0.159) | (0.29) | (0.165) | (0.30) | (0.179) | (0.30) | (0.083) | (0.30) | (0.089) |
| ocountry33 | (0.20) | (0.526) | (0.21) | (0.547) | (0.21) | (0.512) | (0.21) | (0.647) | (0.21) | (0.631) | (0.23) | (0.849) | (0.23) | (0.870) |
| ocountry34 | (1.01) | (0.138) | (1.01) | (0.143) | (1.01) | (0.144) | (1.01) | (0.160) | (1.01) | (0.160) | (1.01) | (0.142) | (1.01) | (0.140) |
| ocountry35 | (0.13) | (0.010) | (0.13) | (0.012) | (0.13) | (0.008) | (0.13) | (0.001) | (0.13) | (0.001) | (0.13) | (0.001) | (0.13) | (0.001) |
| ocountry36 | (0.30) | (0.004) | (0.30) | (0.004) | (0.33) | (0.002) | (0.33) | (0.003) | (0.33) | (0.003) | (0.33) | (0.005) | (0.33) | (0.005) |
| ocountry37 | (0.45) | (0.608) | (0.45) | (0.611) | (0.45) | (0.621) | (0.45) | (0.654) | (0.46) | (0.662) | (0.46) | (0.536) | (0.46) | (0.549) |
| ocountry38 | (0.19) | (0.519) | (0.19) | (0.450) | (0.19) | (0.377) | (0.19) | (0.609) | (0.19) | (0.604) | (0.20) | (0.558) | (0.20) | (0.550) |
| ocountry39 | (0.66) | (0.923) | (0.66) | (0.917) | (0.66) | (0.926) | (0.52) | (0.401) | (0.52) | (0.400) | (0.51) | (0.335) | (0.51) | (0.337) |
| ocountry40 | (0.55) | (0.271) | (0.55) | (0.275) | (0.55) | (0.279) | (0.55) | (0.278) | (0.55) | (0.277) | (0.55) | (0.362) | (0.55) | (0.354) |
| ocountry41 | (0.30) | (0.143) | (0.30) | (0.152) | (0.30) | (0.161) | (0.30) | (0.162) | (0.30) | (0.161) | (0.30) | (0.152) | (0.30) | (0.148) |
| ocountry42 | (1.01) | (0.232) | (1.01) | (0.233) | (1.01) | (0.237) | (1.01) | (0.268) | (1.01) | (0.267) | (1.02) | (0.211) | (1.02) | (0.212) |
| ocountry43 | (0.27) | (0.550) | (0.27) | (0.542) | (0.27) | (0.472) | (0.27) | (0.444) | (0.27) | (0.434) | (0.27) | (0.551) | (0.27) | (0.537) |
| ocountry44 | (0.67) | (0.522) | (0.67) | (0.532) | (0.67) | (0.534) | (0.66) | (0.531) | (0.67) | (0.542) | (0.67) | (0.437) | (0.67) | (0.445) |
| ocountry45 | (0.39) | (0.094) | (0.39) | (0.089) | (0.39) | (0.087) | (0.38) | (0.077) | (0.38) | (0.074) | (0.38) | (0.093) | (0.38) | (0.095) |
| ocountry46 | (0.18) | (0.004) | (0.18) | (0.004) | (0.18) | (0.003) | (0.19) | (0.002) | (0.19) | (0.002) | (0.19) | (0.007) | (0.19) | (0.008) |
| ocountry47 | (0.10) | (0.047) | (0.10) | (0.058) | (0.10) | (0.087) | (0.10) | (0.067) | (0.10) | (0.065) | (0.11) | (0.086) | (0.11) | (0.093) |
| Model ID | 8 |  | 9 |  | 10 |  | 11 |  | 12 |  | 13 |  | 14 |  |
| Variable name | pfr |  | 25Mar |  | 26Mar |  | 27Mar |  | 28Mar |  | 2Apr |  | 7Apr |  |
| ocountry1 | (0.35) | (0.456) | (0.35) | (0.448) | (0.35) | (0.706) | (0.36) | (0.447) | (0.36) | (0.497) | (0.36) | (0.152) | (0.36) | (0.181) |
| ocountry2 | (0.38) | (0.000) | (0.38) | (0.000) | (0.38) | (0.000) | (0.38) | (0.000) | (0.38) | (0.000) | (0.39) | (0.000) | (0.39) | (0.000) |
| ocountry4 | (0.51) | (0.483) | (0.51) | (0.477) | (0.51) | (0.227) | (0.57) | (0.288) | (0.56) | (0.288) | (0.57) | (0.405) | (0.57) | (0.380) |
| ocountry5 | (0.38) | (0.023) | (0.38) | (0.025) | (0.38) | (0.017) | (0.38) | (0.012) | (0.38) | (0.013) | (0.38) | (0.019) | (0.38) | (0.019) |
| ocountry6 | (0.29) | (0.365) | (0.29) | (0.359) | (0.30) | (0.333) | (0.30) | (0.298) | (0.30) | (0.308) | (0.30) | (0.246) | (0.30) | (0.248) |
| ocountry7 | (0.37) | (0.725) | (0.37) | (0.742) | (0.37) | (0.783) | (0.39) | (0.484) | (0.38) | (0.491) | (0.39) | (0.882) | (0.39) | (0.855) |
| ocountry8 | (0.36) | (0.454) | (0.36) | (0.458) | (0.37) | (0.828) | (0.36) | (0.701) | (0.36) | (0.655) | (0.37) | (0.988) | (0.36) | (0.974) |
| ocountry9 | (0.23) | (0.222) | (0.23) | (0.222) | (0.23) | (0.027) | (0.23) | (0.028) | (0.23) | (0.035) | (0.23) | (0.028) | (0.23) | (0.029) |
| ocountry10 | (0.59) | (0.761) | (0.59) | (0.767) | (0.59) | (0.741) | (0.60) | (0.999) | (0.60) | (0.969) | (0.60) | (0.946) | (0.60) | (0.940) |
| ocountry11 | (0.43) | (0.096) | (0.43) | (0.098) | (0.42) | (0.049) | (0.43) | (0.112) | (0.43) | (0.096) | (0.44) | (0.292) | (0.44) | (0.258) |
| ocountry13 | (0.57) | (0.214) | (0.57) | (0.216) | (0.57) | (0.191) | (0.58) | (0.344) | (0.58) | (0.317) | (0.59) | (0.390) | (0.59) | (0.372) |
| ocountry14 | (0.15) | (0.286) | (0.15) | (0.267) | (0.15) | (0.098) | (0.15) | (0.052) | (0.15) | (0.050) | (0.15) | (0.216) | (0.15) | (0.197) |
| ocountry15 | (0.46) | (0.126) | (0.46) | (0.127) | (0.46) | (0.071) | (0.46) | (0.072) | (0.46) | (0.074) | (0.46) | (0.100) | (0.46) | (0.101) |
| ocountry17 | (0.83) | (0.715) | (0.83) | (0.689) | (0.84) | (0.786) | (0.84) | (0.842) | (0.83) | (0.815) | (0.83) | (0.706) | (0.83) | (0.717) |
| ocountry18 | (0.39) | (0.431) | (0.39) | (0.435) | (0.39) | (0.220) | (0.39) | (0.254) | (0.46) | (0.545) | (0.43) | (0.176) | (0.43) | (0.180) |
| ocountry19 | (0.35) | (0.159) | (0.35) | (0.161) | (0.37) | (0.726) | (0.37) | (0.702) | (0.37) | (0.685) | (0.37) | (0.908) | (0.37) | (0.897) |
| ocountry20 | (0.51) | (0.377) | (0.51) | (0.384) | (0.51) | (0.330) | (0.51) | (0.239) | (0.51) | (0.241) | (0.51) | (0.465) | (0.51) | (0.452) |
| ocountry21 | (0.26) | (0.141) | (0.26) | (0.151) | (0.26) | (0.794) | (0.26) | (0.957) | (0.26) | (0.950) | (0.26) | (0.370) | (0.26) | (0.426) |
| ocountry22 | (0.46) | (0.313) | (0.46) | (0.305) | (0.46) | (0.141) | (0.46) | (0.127) | (0.46) | (0.121) | (0.45) | (0.413) | (0.45) | (0.546) |
| ocountry24 | (0.46) | (0.599) | (0.46) | (0.592) | (0.46) | (0.749) | (0.47) | (0.514) | (0.46) | (0.549) | (0.47) | (0.319) | (0.47) | (0.348) |
| ocountry25 | (0.45) | (0.496) | (0.45) | (0.501) | (0.46) | (0.800) | (0.47) | (0.673) | (0.47) | (0.655) | (0.46) | (0.707) | (0.46) | (0.705) |
| ocountry26 | (1.03) | (0.113) | (1.03) | (0.113) | (1.03) | (0.162) | (1.03) | (0.154) | (1.03) | (0.140) | (1.02) | (0.299) | (1.02) | (0.287) |
| ocountry27 | (0.56) | (0.000) | (0.56) | (0.000) | (0.54) | (0.000) | (0.56) | (0.000) | (0.56) | (0.000) | (0.54) | (0.000) | (0.54) | (0.000) |
| ocountry28 | (0.50) | (0.671) | (0.50) | (0.683) | (0.50) | (0.880) | (0.50) | (0.878) | (0.50) | (0.881) | (0.50) | (0.723) | (0.50) | (0.740) |
| ocountry29 | (0.35) | (0.006) | (0.35) | (0.006) | (0.35) | (0.007) | (0.37) | (0.002) | (0.37) | (0.002) | (0.37) | (0.002) | (0.37) | (0.003) |
| ocountry30 | (0.20) | (0.015) | (0.20) | (0.014) | (0.20) | (0.043) | (0.22) | (0.013) | (0.22) | (0.016) | (0.22) | (0.003) | (0.22) | (0.004) |
| ocountry32 | (0.30) | (0.089) | (0.30) | (0.090) | (0.30) | (0.089) | (0.32) | (0.315) | (0.32) | (0.281) | (0.33) | (0.221) | (0.33) | (0.221) |
| ocountry33 | (0.23) | (0.858) | (0.23) | (0.886) | (0.23) | (0.702) | (0.24) | (0.391) | (0.24) | (0.397) | (0.23) | (0.884) | (0.23) | (0.970) |
| ocountry34 | (1.01) | (0.141) | (1.01) | (0.143) | (1.02) | (0.294) | (1.02) | (0.302) | (1.02) | (0.301) | (1.02) | (0.298) | (1.02) | (0.298) |
| ocountry35 | (0.13) | (0.001) | (0.13) | (0.001) | (0.13) | (0.001) | (0.12) | (0.001) | (0.13) | (0.000) | (0.12) | (0.000) | (0.12) | (0.000) |
| ocountry36 | (0.34) | (0.007) | (0.33) | (0.008) | (0.34) | (0.002) | (0.34) | (0.001) | (0.34) | (0.001) | (0.33) | (0.009) | (0.33) | (0.007) |
| ocountry37 | (0.46) | (0.557) | (0.46) | (0.561) | (0.46) | (0.555) | (0.47) | (0.439) | (0.47) | (0.419) | (0.47) | (0.852) | (0.47) | (0.815) |
| ocountry38 | (0.20) | (0.527) | (0.20) | (0.560) | (0.20) | (0.783) | (0.20) | (0.932) | (0.20) | (0.977) | (0.20) | (0.413) | (0.20) | (0.454) |
| ocountry39 | (0.51) | (0.335) | (0.51) | (0.335) | (0.50) | (0.405) | (0.49) | (0.488) | (0.49) | (0.491) | (0.48) | (0.366) | (0.48) | (0.356) |
| ocountry40 | (0.55) | (0.355) | (0.55) | (0.359) | (0.55) | (0.389) | (0.55) | (0.414) | (0.55) | (0.414) | (0.55) | (0.472) | (0.54) | (0.465) |
| ocountry41 | (0.30) | (0.147) | (0.30) | (0.152) | (0.30) | (0.240) | (0.30) | (0.268) | (0.30) | (0.275) | (0.30) | (0.208) | (0.30) | (0.211) |
| ocountry42 | (1.03) | (0.223) | (1.03) | (0.220) | (1.03) | (0.305) | (1.03) | (0.297) | (1.04) | (0.272) | (1.03) | (0.480) | (1.03) | (0.471) |
| ocountry43 | (0.28) | (0.506) | (0.29) | (0.362) | (0.28) | (0.378) | (0.27) | (0.247) | (0.27) | (0.263) | (0.26) | (0.157) | (0.26) | (0.136) |
| ocountry44 | (0.67) | (0.447) | (0.67) | (0.453) | (0.67) | (0.422) | (0.68) | (0.608) | (0.67) | (0.581) | (0.68) | (0.610) | (0.68) | (0.598) |
| ocountry45 | (0.38) | (0.094) | (0.38) | (0.090) | (0.38) | (0.148) | (0.39) | (0.085) | (0.38) | (0.091) | (0.39) | (0.060) | (0.39) | (0.061) |
| ocountry46 | (0.20) | (0.009) | (0.20) | (0.009) | (0.19) | (0.001) | (0.20) | (0.000) | (0.20) | (0.001) | (0.20) | (0.000) | (0.20) | (0.000) |
| ocountry47 | (0.11) | (0.095) | (0.11) | (0.096) | (0.10) | (0.065) | (0.10) | (0.063) | (0.10) | (0.059) | (0.10) | (0.385) | (0.10) | (0.345) |
| N=observation | 1176 |  | 1176 |  | 1176 |  | 1176 |  | 1176 |  | 1176 |  | 1176 |  |

|  |  |  |  |  |  |  |  |  |  |  |  |  |  |  |  |
| --- | --- | --- | --- | --- | --- | --- | --- | --- | --- | --- | --- | --- | --- | --- | --- |
| Model ID | 1 |  |  | 2 |  |  | 3 |  |  | 4 |  |  | 5 |  |  |
|  | 11Mar |  |  | 13Mar |  |  | 16Mar |  |  | 17Mar |  |  | 19Mar |  |  |
| variable name | MEM | [95% Conf. Interval] | | MEM | [95% Conf. Interval] | | MEM | [95% Conf. Interval] | | MEM | [95% Conf. Interval] | | MEM | [95% Conf. Interval] | |
| ocountry1 | 0.29 | [-0.35 | 0.92] | 0.30 | [-0.33 | 0.94] | 0.30 | [-0.33 | 0.93] | 0.30 | [-0.32 | 0.93] | 0.31 | [-0.32 | 0.94] |
| ocountry2 | 1.38*** | [0.63 | 2.12] | 1.37*** | [0.62 | 2.12] | 1.38*** | [0.64 | 2.13] | 1.38*** | [0.65 | 2.12] | 1.39*** | [0.65 | 2.14] |
| ocountry4 | -0.08 | [-1.08 | 0.93] | -0.09 | [-1.10 | 0.91] | -0.07 | [-1.08 | 0.93] | 0.30 | [-0.70 | 1.29] | 0.31 | [-0.69 | 1.30] |
| ocountry5 | 0.74** | [0.00 | 1.48] | 0.74* | [-0.00 | 1.47] | 0.81** | [0.06 | 1.56] | 0.88** | [0.14 | 1.62] | 0.88** | [0.14 | 1.62] |
| ocountry6 | 0.16 | [-0.42 | 0.73] | 0.16 | [-0.41 | 0.74] | 0.18 | [-0.40 | 0.75] | 0.23 | [-0.36 | 0.81] | 0.23 | [-0.36 | 0.81] |
| ocountry7 | -0.05 | [-0.78 | 0.67] | -0.05 | [-0.77 | 0.68] | -0.04 | [-0.76 | 0.69] | -0.01 | [-0.73 | 0.71] | -0.00 | [-0.73 | 0.72] |
| ocountry8 | -0.34 | [-1.03 | 0.36] | -0.34 | [-1.04 | 0.36] | -0.33 | [-1.03 | 0.37] | -0.33 | [-1.02 | 0.37] | -0.32 | [-1.01 | 0.38] |
| ocountry9 | 0.28 | [-0.16 | 0.71] | 0.27 | [-0.16 | 0.71] | 0.29 | [-0.14 | 0.73] | 0.31 | [-0.13 | 0.75] | 0.31 | [-0.13 | 0.75] |
| ocountry10 | -0.10 | [-1.26 | 1.06] | -0.09 | [-1.25 | 1.07] | -0.08 | [-1.24 | 1.08] | -0.08 | [-1.23 | 1.07] | -0.07 | [-1.22 | 1.09] |
| ocountry11 | -0.68* | [-1.48 | 0.12] | -0.67 | [-1.47 | 0.13] | -0.67 | [-1.47 | 0.13] | -0.67* | [-1.46 | 0.13] | -0.66 | [-1.45 | 0.14] |
| ocountry13 | -0.58 | [-1.69 | 0.52] | -0.58 | [-1.69 | 0.53] | -0.58 | [-1.68 | 0.53] | -0.59 | [-1.69 | 0.51] | -0.58 | [-1.68 | 0.52] |
| ocountry14 | 0.13 | [-0.15 | 0.40] | 0.09 | [-0.19 | 0.38] | 0.09 | [-0.19 | 0.38] | 0.15 | [-0.13 | 0.44] | 0.15 | [-0.13 | 0.44] |
| ocountry15 | -0.57 | [-1.45 | 0.31] | -0.67 | [-1.58 | 0.24] | -0.66 | [-1.57 | 0.25] | -0.60 | [-1.51 | 0.31] | -0.60 | [-1.51 | 0.31] |
| ocountry17 | -0.36 | [-1.98 | 1.25] | -0.37 | [-1.98 | 1.25] | -0.24 | [-1.86 | 1.39] | -0.24 | [-1.86 | 1.39] | -0.24 | [-1.86 | 1.39] |
| ocountry18 | 0.30 | [-0.42 | 1.02] | 0.31 | [-0.42 | 1.03] | 0.32 | [-0.41 | 1.04] | 0.43 | [-0.30 | 1.16] | 0.43 | [-0.30 | 1.16] |
| ocountry19 | -0.54 | [-1.21 | 0.14] | -0.52 | [-1.20 | 0.15] | -0.52 | [-1.19 | 0.16] | -0.44 | [-1.14 | 0.25] | -0.45 | [-1.14 | 0.25] |
| ocountry20 | -0.46 | [-1.45 | 0.53] | -0.45 | [-1.44 | 0.53] | -0.45 | [-1.44 | 0.54] | -0.43 | [-1.41 | 0.56] | -0.42 | [-1.41 | 0.57] |
| ocountry21 | -0.32 | [-0.78 | 0.13] | -0.32 | [-0.78 | 0.14] | -0.30 | [-0.76 | 0.15] | -0.28 | [-0.73 | 0.18] | -0.28 | [-0.73 | 0.18] |
| ocountry22 | -0.65 | [-1.52 | 0.22] | -0.65 | [-1.52 | 0.22] | -0.63 | [-1.50 | 0.24] | -0.56 | [-1.44 | 0.32] | -0.56 | [-1.44 | 0.32] |
| ocountry24 | 0.28 | [-0.59 | 1.16] | 0.30 | [-0.58 | 1.17] | 0.30 | [-0.58 | 1.17] | 0.30 | [-0.57 | 1.17] | 0.31 | [-0.56 | 1.18] |
| ocountry25 | -0.27 | [-1.15 | 0.61] | -0.28 | [-1.16 | 0.60] | -0.27 | [-1.15 | 0.61] | -0.27 | [-1.15 | 0.61] | -0.26 | [-1.15 | 0.62] |
| ocountry26 | -1.63 | [-3.61 | 0.35] | -1.61 | [-3.59 | 0.37] | -1.61 | [-3.59 | 0.37] | -1.56 | [-3.54 | 0.42] | -1.56 | [-3.54 | 0.42] |
| ocountry27 | 0.91** | [0.06 | 1.77] | 0.91** | [0.05 | 1.76] | 0.91** | [0.05 | 1.76] | 2.31*** | [1.20 | 3.42] | 2.31*** | [1.19 | 3.42] |
| ocountry28 | -0.26 | [-1.24 | 0.72] | -0.25 | [-1.23 | 0.73] | -0.24 | [-1.22 | 0.74] | -0.19 | [-1.18 | 0.80] | -0.19 | [-1.18 | 0.80] |
| ocountry29 | 1.05*** | [0.37 | 1.72] | 1.06*** | [0.38 | 1.73] | 1.06*** | [0.38 | 1.74] | 1.06*** | [0.40 | 1.73] | 1.08*** | [0.40 | 1.75] |
| ocountry30 | 0.52*** | [0.14 | 0.89] | 0.53*** | [0.15 | 0.91] | 0.53*** | [0.15 | 0.91] | 0.54*** | [0.16 | 0.91] | 0.55*** | [0.16 | 0.93] |
| ocountry32 | -0.43 | [-1.01 | 0.14] | -0.43 | [-1.01 | 0.15] | -0.41 | [-0.99 | 0.16] | -0.41 | [-0.98 | 0.17] | -0.40 | [-0.97 | 0.18] |
| ocountry33 | 0.13 | [-0.27 | 0.53] | 0.12 | [-0.28 | 0.53] | 0.13 | [-0.27 | 0.54] | 0.09 | [-0.31 | 0.50] | 0.10 | [-0.30 | 0.50] |
| ocountry34 | -1.49 | [-3.46 | 0.48] | -1.48 | [-3.45 | 0.50] | -1.47 | [-3.44 | 0.50] | -1.42 | [-3.39 | 0.56] | -1.42 | [-3.39 | 0.56] |
| ocountry35 | 0.32** | [0.08 | 0.57] | 0.32** | [0.07 | 0.56] | 0.33*** | [0.09 | 0.58] | 0.42*** | [0.16 | 0.67] | 0.42*** | [0.17 | 0.68] |
| ocountry36 | 0.87*** | [0.27 | 1.47] | 0.86*** | [0.27 | 1.46] | 1.00*** | [0.36 | 1.64] | 0.98*** | [0.34 | 1.62] | 0.99*** | [0.35 | 1.63] |
| ocountry37 | -0.23 | [-1.12 | 0.66] | -0.23 | [-1.12 | 0.66] | -0.22 | [-1.11 | 0.66] | -0.20 | [-1.10 | 0.69] | -0.20 | [-1.09 | 0.69] |
| ocountry38 | -0.12 | [-0.49 | 0.25] | -0.14 | [-0.52 | 0.23] | -0.17 | [-0.54 | 0.21] | -0.10 | [-0.47 | 0.28] | -0.10 | [-0.47 | 0.27] |
| ocountry39 | -0.06 | [-1.35 | 1.22] | -0.07 | [-1.36 | 1.22] | -0.06 | [-1.35 | 1.23] | -0.44 | [-1.45 | 0.58] | -0.44 | [-1.45 | 0.58] |
| ocountry40 | -0.61 | [-1.69 | 0.48] | -0.60 | [-1.69 | 0.48] | -0.60 | [-1.68 | 0.48] | -0.60 | [-1.68 | 0.48] | -0.60 | [-1.68 | 0.48] |
| ocountry41 | -0.43 | [-1.01 | 0.15] | -0.42 | [-1.00 | 0.16] | -0.41 | [-0.99 | 0.17] | -0.42 | [-1.00 | 0.17] | -0.42 | [-1.00 | 0.17] |
| ocountry42 | -1.21 | [-3.19 | 0.77] | -1.20 | [-3.18 | 0.77] | -1.19 | [-3.17 | 0.79] | -1.12 | [-3.11 | 0.86] | -1.12 | [-3.11 | 0.86] |
| ocountry43 | 0.16 | [-0.37 | 0.69] | 0.17 | [-0.37 | 0.70] | 0.20 | [-0.34 | 0.73] | 0.21 | [-0.33 | 0.75] | 0.21 | [-0.32 | 0.75] |
| ocountry44 | -0.43 | [-1.74 | 0.88] | -0.42 | [-1.73 | 0.90] | -0.42 | [-1.73 | 0.90] | -0.42 | [-1.72 | 0.89] | -0.41 | [-1.71 | 0.90] |
| ocountry45 | 0.65* | [-0.11 | 1.40] | 0.66* | [-0.10 | 1.42] | 0.66* | [-0.10 | 1.42] | 0.68* | [-0.07 | 1.42] | 0.68* | [-0.07 | 1.44] |
| ocountry46 | 0.53*** | [0.17 | 0.89] | 0.53*** | [0.17 | 0.89] | 0.54*** | [0.18 | 0.90] | 0.59*** | [0.22 | 0.95] | 0.59*** | [0.23 | 0.96] |
| ocountry47 | -0.20** | [-0.40 | -0.00] | -0.19* | [-0.40 | 0.01] | -0.18* | [-0.38 | 0.03] | -0.19* | [-0.39 | 0.01] | -0.19* | [-0.40 | 0.01] |
| Model ID | 6 |  |  | 7 |  |  | 8 |  |  | 9 |  |  | 10 |  |  |
|  | pfl |  |  | 21Mar |  |  | pfr |  |  | 25Mar |  |  | 26Mar |  |  |
| variable name | MEM | [95% Conf. Interval] | | MEM | [95% Conf. Interval] | | MEM | [95% Conf. Interval] | | MEM | [95% Conf. Interval] | | MEM | [95% Conf. Interval] | |
| ocountry1 | 0.25 | [-0.43 | 0.93] | 0.25 | [-0.43 | 0.94] | 0.26 | [-0.43 | 0.95] | 0.27 | [-0.42 | 0.96] | 0.13 | [-0.55 | 0.81] |
| ocountry2 | 1.48*** | [0.74 | 2.22] | 1.49*** | [0.74 | 2.23] | 1.49*** | [0.74 | 2.23] | 1.49*** | [0.74 | 2.24] | 1.68*** | [0.93 | 2.43] |
| ocountry4 | 0.35 | [-0.64 | 1.34] | 0.36 | [-0.64 | 1.35] | 0.36 | [-0.64 | 1.35] | 0.36 | [-0.64 | 1.36] | 0.62 | [-0.39 | 1.62] |
| ocountry5 | 0.86** | [0.12 | 1.60] | 0.87** | [0.13 | 1.61] | 0.86** | [0.12 | 1.60] | 0.85** | [0.11 | 1.58] | 0.90** | [0.16 | 1.64] |
| ocountry6 | 0.27 | [-0.30 | 0.84] | 0.26 | [-0.31 | 0.83] | 0.27 | [-0.31 | 0.84] | 0.27 | [-0.31 | 0.84] | 0.29 | [-0.30 | 0.89] |
| ocountry7 | -0.13 | [-0.85 | 0.60] | -0.13 | [-0.85 | 0.60] | -0.13 | [-0.86 | 0.60] | -0.12 | [-0.85 | 0.60] | 0.10 | [-0.63 | 0.84] |
| ocountry8 | -0.29 | [-0.99 | 0.42] | -0.28 | [-0.99 | 0.43] | -0.27 | [-0.98 | 0.44] | -0.27 | [-0.98 | 0.44] | -0.08 | [-0.80 | 0.64] |
| ocountry9 | 0.27 | [-0.17 | 0.71] | 0.28 | [-0.17 | 0.72] | 0.28 | [-0.17 | 0.72] | 0.28 | [-0.17 | 0.72] | 0.50** | [0.06 | 0.95] |
| ocountry10 | -0.19 | [-1.34 | 0.97] | -0.18 | [-1.34 | 0.98] | -0.18 | [-1.34 | 0.98] | -0.18 | [-1.34 | 0.99] | -0.19 | [-1.35 | 0.96] |
| ocountry11 | -0.72* | [-1.55 | 0.11] | -0.72* | [-1.55 | 0.12] | -0.71* | [-1.55 | 0.13] | -0.71* | [-1.55 | 0.13] | -0.83** | [-1.67 | -0.00] |
| ocountry13 | -0.73 | [-1.84 | 0.39] | -0.71 | [-1.83 | 0.41] | -0.71 | [-1.83 | 0.41] | -0.71 | [-1.83 | 0.41] | -0.75 | [-1.86 | 0.37] |
| ocountry14 | 0.16 | [-0.12 | 0.45] | 0.16 | [-0.13 | 0.45] | 0.16 | [-0.13 | 0.45] | 0.16 | [-0.13 | 0.45] | 0.25* | [-0.05 | 0.54] |
| ocountry15 | -0.70 | [-1.61 | 0.21] | -0.71 | [-1.62 | 0.20] | -0.71 | [-1.62 | 0.20] | -0.71 | [-1.62 | 0.20] | -0.84* | [-1.74 | 0.07] |
| ocountry17 | -0.30 | [-1.92 | 1.31] | -0.28 | [-1.91 | 1.34] | -0.30 | [-1.93 | 1.33] | -0.33 | [-1.96 | 1.30] | -0.23 | [-1.86 | 1.41] |
| ocountry18 | 0.30 | [-0.44 | 1.05] | 0.30 | [-0.44 | 1.04] | 0.31 | [-0.46 | 1.07] | 0.30 | [-0.46 | 1.07] | 0.47 | [-0.28 | 1.23] |
| ocountry19 | -0.49 | [-1.18 | 0.20] | -0.50 | [-1.19 | 0.19] | -0.50 | [-1.19 | 0.19] | -0.50 | [-1.19 | 0.20] | -0.13 | [-0.85 | 0.59] |
| ocountry20 | -0.45 | [-1.45 | 0.54] | -0.45 | [-1.44 | 0.55] | -0.45 | [-1.44 | 0.55] | -0.44 | [-1.44 | 0.55] | -0.49 | [-1.49 | 0.50] |
| ocountry21 | -0.37 | [-0.87 | 0.13] | -0.37 | [-0.87 | 0.13] | -0.38 | [-0.88 | 0.12] | -0.37 | [-0.87 | 0.13] | -0.07 | [-0.57 | 0.43] |
| ocountry22 | -0.47 | [-1.37 | 0.43] | -0.47 | [-1.36 | 0.43] | -0.46 | [-1.36 | 0.44] | -0.47 | [-1.37 | 0.43] | -0.68 | [-1.58 | 0.22] |
| ocountry24 | 0.23 | [-0.66 | 1.12] | 0.24 | [-0.66 | 1.13] | 0.24 | [-0.66 | 1.14] | 0.25 | [-0.65 | 1.15] | 0.15 | [-0.75 | 1.04] |
| ocountry25 | -0.32 | [-1.20 | 0.56] | -0.30 | [-1.19 | 0.58] | -0.31 | [-1.19 | 0.58] | -0.30 | [-1.19 | 0.58] | -0.12 | [-1.02 | 0.79] |
| ocountry26 | -1.64 | [-3.64 | 0.35] | -1.65 | [-3.64 | 0.35] | -1.63 | [-3.64 | 0.38] | -1.63 | [-3.64 | 0.39] | -1.44 | [-3.45 | 0.58] |
| ocountry27 | 2.26*** | [1.16 | 3.35] | 2.25*** | [1.16 | 3.34] | 2.25*** | [1.16 | 3.35] | 2.26*** | [1.17 | 3.35] | 2.18*** | [1.13 | 3.24] |
| ocountry28 | -0.21 | [-1.20 | 0.78] | -0.22 | [-1.20 | 0.77] | -0.21 | [-1.20 | 0.77] | -0.21 | [-1.19 | 0.78] | 0.08 | [-0.91 | 1.06] |
| ocountry29 | 0.97*** | [0.28 | 1.65] | 0.97*** | [0.28 | 1.66] | 0.97*** | [0.28 | 1.66] | 0.98*** | [0.28 | 1.67] | 0.95*** | [0.26 | 1.64] |
| ocountry30 | 0.49** | [0.09 | 0.88] | 0.49** | [0.09 | 0.88] | 0.49** | [0.09 | 0.89] | 0.50** | [0.10 | 0.90] | 0.41** | [0.01 | 0.80] |
| ocountry32 | -0.52* | [-1.12 | 0.07] | -0.52* | [-1.11 | 0.08] | -0.52* | [-1.11 | 0.08] | -0.52* | [-1.11 | 0.08] | -0.52* | [-1.11 | 0.08] |
| ocountry33 | -0.04 | [-0.48 | 0.40] | -0.04 | [-0.48 | 0.40] | -0.04 | [-0.48 | 0.40] | -0.03 | [-0.48 | 0.41] | 0.09 | [-0.36 | 0.53] |
| ocountry34 | -1.48 | [-3.46 | 0.50] | -1.49 | [-3.47 | 0.49] | -1.49 | [-3.46 | 0.49] | -1.48 | [-3.46 | 0.50] | -1.07 | [-3.06 | 0.93] |
| ocountry35 | 0.45*** | [0.20 | 0.70] | 0.45*** | [0.19 | 0.70] | 0.45*** | [0.19 | 0.70] | 0.45*** | [0.19 | 0.70] | 0.40*** | [0.16 | 0.65] |
| ocountry36 | 0.91*** | [0.27 | 1.55] | 0.92*** | [0.28 | 1.57] | 0.90*** | [0.24 | 1.56] | 0.88*** | [0.23 | 1.54] | 1.05*** | [0.38 | 1.71] |
| ocountry37 | -0.29 | [-1.19 | 0.62] | -0.28 | [-1.18 | 0.63] | -0.27 | [-1.18 | 0.64] | -0.27 | [-1.18 | 0.64] | -0.27 | [-1.18 | 0.64] |
| ocountry38 | -0.12 | [-0.50 | 0.27] | -0.12 | [-0.50 | 0.27] | -0.13 | [-0.52 | 0.26] | -0.12 | [-0.51 | 0.28] | -0.05 | [-0.44 | 0.33] |
| ocountry39 | -0.49 | [-1.50 | 0.51] | -0.49 | [-1.49 | 0.51] | -0.49 | [-1.50 | 0.51] | -0.49 | [-1.50 | 0.51] | -0.41 | [-1.38 | 0.56] |
| ocountry40 | -0.50 | [-1.57 | 0.57] | -0.51 | [-1.58 | 0.57] | -0.51 | [-1.58 | 0.57] | -0.50 | [-1.58 | 0.57] | -0.47 | [-1.54 | 0.60] |
| ocountry41 | -0.43 | [-1.01 | 0.16] | -0.43 | [-1.01 | 0.15] | -0.43 | [-1.02 | 0.15] | -0.43 | [-1.01 | 0.16] | -0.35 | [-0.95 | 0.24] |
| ocountry42 | -1.27 | [-3.27 | 0.72] | -1.27 | [-3.27 | 0.73] | -1.26 | [-3.28 | 0.76] | -1.27 | [-3.29 | 0.76] | -1.06 | [-3.09 | 0.96] |
| ocountry43 | 0.16 | [-0.37 | 0.70] | 0.17 | [-0.37 | 0.70] | 0.19 | [-0.36 | 0.73] | 0.26 | [-0.30 | 0.82] | 0.24 | [-0.30 | 0.78] |
| ocountry44 | -0.52 | [-1.83 | 0.79] | -0.51 | [-1.82 | 0.80] | -0.51 | [-1.82 | 0.80] | -0.50 | [-1.82 | 0.81] | -0.54 | [-1.84 | 0.77] |
| ocountry45 | 0.64* | [-0.11 | 1.38] | 0.64* | [-0.11 | 1.39] | 0.64* | [-0.11 | 1.39] | 0.65* | [-0.10 | 1.40] | 0.55 | [-0.20 | 1.30] |
| ocountry46 | 0.51*** | [0.14 | 0.89] | 0.51*** | [0.13 | 0.89] | 0.51*** | [0.13 | 0.90] | 0.51*** | [0.13 | 0.90] | 0.60*** | [0.23 | 0.98] |
| ocountry47 | -0.18* | [-0.39 | 0.03] | -0.18* | [-0.38 | 0.03] | -0.18* | [-0.38 | 0.03] | -0.18* | [-0.38 | 0.03] | -0.19* | [-0.39 | 0.01] |
| Model ID | 11 |  |  | 12 |  |  | 13 |  |  | 14 |  |  |  |  |  |
|  | 27Mar |  |  | 28Mar |  |  | 2Apr |  |  | 7Apr |  |  |  |  |  |
| variable name | MEM | [95% Conf. Interval] | | MEM | [95% Conf. Interval] | | MEM | [95% Conf. Interval] | | MEM | [95% Conf. Interval] | |  |  |  |
| ocountry1 | 0.27 | [-0.43 | 0.98] | 0.24 | [-0.46 | 0.95] | 0.52 | [-0.19 | 1.23] | 0.49 | [-0.23 | 1.20] |  |  |  |
| ocountry2 | 1.61*** | [0.86 | 2.36] | 1.60*** | [0.86 | 2.34] | 1.63*** | [0.88 | 2.39] | 1.63*** | [0.87 | 2.38] |  |  |  |
| ocountry4 | 0.60 | [-0.51 | 1.71] | 0.60 | [-0.51 | 1.70] | 0.47 | [-0.64 | 1.59] | 0.50 | [-0.61 | 1.61] |  |  |  |
| ocountry5 | 0.95** | [0.21 | 1.69] | 0.95** | [0.20 | 1.70] | 0.89** | [0.14 | 1.63] | 0.89** | [0.15 | 1.63] |  |  |  |
| ocountry6 | 0.32 | [-0.28 | 0.91] | 0.31 | [-0.29 | 0.90] | 0.35 | [-0.24 | 0.94] | 0.35 | [-0.24 | 0.94] |  |  |  |
| ocountry7 | 0.27 | [-0.49 | 1.03] | 0.26 | [-0.49 | 1.02] | 0.06 | [-0.70 | 0.81] | 0.07 | [-0.69 | 0.83] |  |  |  |
| ocountry8 | -0.14 | [-0.85 | 0.57] | -0.16 | [-0.87 | 0.55] | 0.01 | [-0.71 | 0.72] | -0.01 | [-0.73 | 0.70] |  |  |  |
| ocountry9 | 0.50** | [0.05 | 0.94] | 0.48** | [0.03 | 0.93] | 0.50** | [0.05 | 0.95] | 0.50** | [0.05 | 0.95] |  |  |  |
| ocountry10 | -0.00 | [-1.18 | 1.18] | -0.02 | [-1.19 | 1.14] | -0.04 | [-1.22 | 1.14] | -0.05 | [-1.23 | 1.14] |  |  |  |
| ocountry11 | -0.69 | [-1.54 | 0.16] | -0.72* | [-1.56 | 0.13] | -0.46 | [-1.32 | 0.40] | -0.49 | [-1.35 | 0.36] |  |  |  |
| ocountry13 | -0.55 | [-1.69 | 0.59] | -0.58 | [-1.72 | 0.56] | -0.50 | [-1.65 | 0.64] | -0.52 | [-1.67 | 0.62] |  |  |  |
| ocountry14 | 0.30* | [-0.00 | 0.59] | 0.30* | [-0.00 | 0.60] | 0.18 | [-0.11 | 0.48] | 0.19 | [-0.10 | 0.49] |  |  |  |
| ocountry15 | -0.84* | [-1.75 | 0.07] | -0.83* | [-1.74 | 0.08] | -0.76 | [-1.67 | 0.15] | -0.76 | [-1.67 | 0.15] |  |  |  |
| ocountry17 | -0.17 | [-1.81 | 1.47] | -0.19 | [-1.83 | 1.44] | -0.31 | [-1.95 | 1.32] | -0.30 | [-1.93 | 1.33] |  |  |  |
| ocountry18 | 0.44 | [-0.32 | 1.21] | 0.28 | [-0.62 | 1.17] | 0.59 | [-0.26 | 1.44] | 0.58 | [-0.27 | 1.43] |  |  |  |
| ocountry19 | -0.14 | [-0.86 | 0.58] | -0.15 | [-0.87 | 0.57] | -0.04 | [-0.76 | 0.68] | -0.05 | [-0.77 | 0.67] |  |  |  |
| ocountry20 | -0.60 | [-1.60 | 0.40] | -0.60 | [-1.60 | 0.40] | -0.37 | [-1.37 | 0.63] | -0.38 | [-1.38 | 0.61] |  |  |  |
| ocountry21 | -0.01 | [-0.52 | 0.49] | -0.02 | [-0.52 | 0.49] | -0.23 | [-0.74 | 0.28] | -0.21 | [-0.71 | 0.30] |  |  |  |
| ocountry22 | -0.70 | [-1.60 | 0.20] | -0.71 | [-1.61 | 0.19] | -0.37 | [-1.26 | 0.52] | -0.27 | [-1.16 | 0.61] |  |  |  |
| ocountry24 | 0.30 | [-0.61 | 1.22] | 0.28 | [-0.63 | 1.18] | 0.47 | [-0.45 | 1.39] | 0.44 | [-0.48 | 1.36] |  |  |  |
| ocountry25 | -0.20 | [-1.11 | 0.72] | -0.21 | [-1.12 | 0.71] | -0.17 | [-1.08 | 0.73] | -0.17 | [-1.08 | 0.73] |  |  |  |
| ocountry26 | -1.47 | [-3.48 | 0.55] | -1.52 | [-3.54 | 0.50] | -1.06 | [-3.07 | 0.94] | -1.09 | [-3.10 | 0.91] |  |  |  |
| ocountry27 | 2.15*** | [1.06 | 3.24] | 2.15*** | [1.06 | 3.24] | 2.21*** | [1.15 | 3.28] | 2.18*** | [1.12 | 3.24] |  |  |  |
| ocountry28 | 0.08 | [-0.91 | 1.06] | 0.08 | [-0.91 | 1.06] | 0.18 | [-0.81 | 1.16] | 0.17 | [-0.82 | 1.15] |  |  |  |
| ocountry29 | 1.14*** | [0.41 | 1.86] | 1.12*** | [0.40 | 1.84] | 1.14*** | [0.40 | 1.87] | 1.12*** | [0.39 | 1.85] |  |  |  |
| ocountry30 | 0.55** | [0.11 | 0.98] | 0.53** | [0.10 | 0.96] | 0.66*** | [0.22 | 1.10] | 0.64*** | [0.20 | 1.08] |  |  |  |
| ocountry32 | -0.33 | [-0.96 | 0.31] | -0.35 | [-0.98 | 0.29] | -0.40 | [-1.04 | 0.24] | -0.40 | [-1.04 | 0.24] |  |  |  |
| ocountry33 | 0.20 | [-0.26 | 0.67] | 0.20 | [-0.26 | 0.66] | -0.03 | [-0.49 | 0.42] | -0.01 | [-0.47 | 0.45] |  |  |  |
| ocountry34 | -1.05 | [-3.04 | 0.94] | -1.05 | [-3.04 | 0.94] | -1.06 | [-3.05 | 0.93] | -1.06 | [-3.05 | 0.93] |  |  |  |
| ocountry35 | 0.43*** | [0.19 | 0.68] | 0.44*** | [0.20 | 0.69] | 0.49*** | [0.24 | 0.73] | 0.48*** | [0.24 | 0.73] |  |  |  |
| ocountry36 | 1.13*** | [0.46 | 1.81] | 1.12*** | [0.46 | 1.79] | 0.88*** | [0.22 | 1.53] | 0.90*** | [0.24 | 1.55] |  |  |  |
| ocountry37 | -0.37 | [-1.29 | 0.56] | -0.38 | [-1.30 | 0.54] | -0.09 | [-1.00 | 0.83] | -0.11 | [-1.02 | 0.81] |  |  |  |
| ocountry38 | -0.02 | [-0.41 | 0.37] | -0.01 | [-0.40 | 0.38] | -0.16 | [-0.54 | 0.22] | -0.15 | [-0.53 | 0.24] |  |  |  |
| ocountry39 | -0.34 | [-1.31 | 0.62] | -0.34 | [-1.30 | 0.62] | -0.44 | [-1.38 | 0.51] | -0.44 | [-1.38 | 0.50] |  |  |  |
| ocountry40 | -0.45 | [-1.51 | 0.62] | -0.45 | [-1.52 | 0.62] | -0.39 | [-1.46 | 0.68] | -0.40 | [-1.47 | 0.67] |  |  |  |
| ocountry41 | -0.34 | [-0.93 | 0.26] | -0.33 | [-0.92 | 0.26] | -0.38 | [-0.98 | 0.21] | -0.38 | [-0.97 | 0.22] |  |  |  |
| ocountry42 | -1.08 | [-3.10 | 0.95] | -1.14 | [-3.17 | 0.89] | -0.73 | [-2.74 | 1.29] | -0.74 | [-2.75 | 1.27] |  |  |  |
| ocountry43 | 0.32 | [-0.22 | 0.85] | 0.31 | [-0.23 | 0.84] | 0.37 | [-0.14 | 0.89] | 0.39 | [-0.12 | 0.90] |  |  |  |
| ocountry44 | -0.35 | [-1.67 | 0.98] | -0.37 | [-1.69 | 0.95] | -0.35 | [-1.68 | 0.99] | -0.36 | [-1.69 | 0.97] |  |  |  |
| ocountry45 | 0.67* | [-0.09 | 1.43] | 0.65* | [-0.10 | 1.40] | 0.74* | [-0.03 | 1.51] | 0.73* | [-0.03 | 1.50] |  |  |  |
| ocountry46 | 0.70*** | [0.31 | 1.09] | 0.68*** | [0.30 | 1.07] | 0.74*** | [0.35 | 1.13] | 0.74*** | [0.34 | 1.13] |  |  |  |
| ocountry47 | -0.19* | [-0.40 | 0.01] | -0.20* | [-0.40 | 0.01] | -0.09 | [-0.29 | 0.11] | -0.10 | [-0.30 | 0.10] |  |  |  |
| N=observation | 1176 |  |  | 1176 |  |  | 1176 |  |  | 1176 |  |  | 1176 |  |  |

Note: (1) **P<0.1, **P < 0.05, ***P < 0.001*; n = 1,176 (effective observations).

(2) The second line of every variable row reports the 95% Confidence interval.

(3) “country3, ocountry12, ocountry16, ocountry23, ocountry31 and ocountry48” were omitted by the regression model due to collinearity.

**SI References**

1. Cohen J. Statistical power analysis for the behavioral sciences. Second. Hillsdale, NJ: L. Erlbaum Associates; 1988. pp. 145–175.
